# Aromatic Residues in Proteins: Re-Evaluating the Geometry and Energetics of π–π, Cation−π, and CH−π Interactions

**DOI:** 10.1021/acs.jpcb.4c04774

**Published:** 2024-09-02

**Authors:** Rivka Calinsky, Yaakov Levy

**Affiliations:** Department of Chemical and Structural Biology, Weizmann Institute of Science, Rehovot 76100, Israel

## Abstract

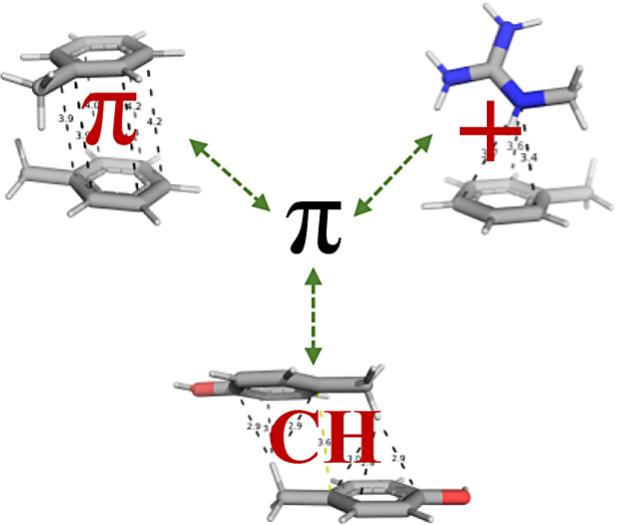

Aromatic residues can participate in various biomolecular interactions, such as π–π, cation−π, and CH−π interactions, which are essential for protein structure and function. Here, we re-evaluate the geometry and energetics of these interactions using quantum mechanical (QM) calculations, focusing on pairwise interactions involving the aromatic amino acids Phe, Tyr, and Trp and the cationic amino acids Arg and Lys. Our findings reveal that π–π interactions, while energetically favorable, are less abundant in structured proteins than commonly assumed and are often overshadowed by previously underappreciated, yet prevalent, CH−π interactions. Cation−π interactions, particularly those involving Arg, show strong binding energies and a specific geometric preference toward stacked conformations, despite the global QM minimum, suggesting that a rather perpendicular T-shape conformation should be more favorable. Our results support a more nuanced understanding of protein stabilization via interactions involving aromatic residues. On the one hand, our results challenge the traditional emphasis on π–π interactions in structured proteins by showing that CH−π and cation−π interactions contribute significantly to their structure. On the other hand, π–π interactions appear to be key stabilizers in solvated regions and thus may be particularly important to the stabilization of intrinsically disordered proteins.

## Introduction

Efforts to understand the variety of interactions occurring within specific amino acid pairs, particularly those involving aromatic side chains, now lie at the forefront of structural biological research and are thus the subject of considerable attention. A diverse range of aromatic side chain interactions play pivotal roles in protein structure and function. Notable among these are π–π interactions (also known as π-stacking and aromatic–aromatic interactions), cation−π interactions, and CH−π interactions.^1−6^ Interactions of the π–π and cation−π types, in particular, are recognized as being key stabilizers governing disordered protein conformations.^7,8^

Π-stacking interactions are enabled by the delocalized π-orbital electrons that characterize aromatic rings, which are found in amino acids bearing aromatic side chains (i.e., Phe, Tyr, Trp, and His). These interactions occur when the planes of two aromatic rings adopt an energetically favorable parallel orientation, referred to as “stacked” conformation, which is often highlighted as stabilizing protein structures.^9−11^ Nevertheless, π-stacked interactions are generally considered as occurring within hydrophobic buried pairs, with binding energies often reported for the gas phase. Our particular interest was drawn to solvent-exposed pairs given their contributions to the properties of intrinsically disordered proteins (IDPs).

Unlike π–π interactions, cation−π interactions involve the side chains of a positively charged cationic amino acid, such as Lys or Arg, and the electron-rich face of an aromatic amino acid. These interactions, although previously underappreciated,^12−15^ have recently gained attention for their substantial role in protein folding and stability.^2,16−19^

Similarly, CH−π interactions, though less studied,^4,20,21^ have emerged as potentially important contributors to the stability of protein structures, following their notable abundance in proteins. Unlike cation−π interactions, which involve a positively charged contribution, these interactions feature a polarized C–H bond, where the hydrogen atom is usually connected to an aromatic ring atom and thus interacts with a negatively charged π-system. CH−π interactions were found to occur, together with other interaction types. For instance, CH−π bonding has been shown to enhance the stability of a specific cation−π interaction.^22^

Notably, the traditional view of π–π interactions as unique stabilizers of protein structures was challenged by evidence suggesting that these interactions may be less crucial than previously thought, with dispersion forces, as well as solvent-driven surface minimization, playing a more dominant role in protein structural stabilization.^23−27^ This perspective, while still evolving, not only challenges long-standing views but also underscores the urgency for a more thorough and sophisticated examination of these molecular interactions. Our research is strategically positioned at this crossroads, aiming to dissect and quantify the relative energetic strengths of various aromatic interactions, namely, π–π, cation−π, CH−π, as well as hydrogen bonds, in the context of protein structure.

Recognizing the significance of π-stacking and cation−π interactions, a variety of computational tools have been developed to identify them, including Arpeggio, RING 2.0, PLIP, Protein Explorer, PIC, CAPTURE, CAD, and CIPDB.^28^ With the exception of CAPTURE, these tools rely solely on geometric definitions. Although CAPTURE incorporates energetic considerations, it only accounts for electrostatics and, to a limited degree, van der Waals interactions but does not rely on dispersion measurements calculated based on coordinates for structures taken from the Protein Data Bank (PDB).^19^

To date, a comprehensive analysis using trustworthy methods that consider solvent effects and compare the strength of π-stacking, CH−π, cation−π, and hydrogen bonding for aromatic amino acids has not been performed. Part of the reason for this is that additive fixed-charge force fields are insufficiently accurate for calculations of the energetics of cation−π or π–π interactions, which include electron delocalization effects.^25^ However, developing a reliable quantum mechanical (QM) method is a far-from-trivial task.^29,30^ Our study aims to fill this gap by evaluating the energetics of various pairwise geometries involving aromatic amino acids, as observed in the published three-dimensional structures of experimentally resolved proteins. Furthermore, we adopt a more integrated perspective that considers not only the energetic contribution but also the prevalence of interactions depending on the exact geometry of the corresponding pairwise interactions. Our results suggest that considering only the single minimal energy conformation to characterize aromatic interactions in proteins may be misleading. We note that this study focuses on aromatic Phe, Tyr, and Trp amino acid residues. His has also aromatic characteristics; however, given that its pH-dependent protonation state determines whether it functions as an aromatic or cationic residue, it will be the focus of a separate work.

## Methods

Our analysis utilized two unique sets of PDB structures to explore aromatic–aromatic and cationic–aromatic amino acid pairs. A primary data set was assembled from PISCES,^31^ drawing on 6535 high-resolution (resolution ≤ 1.8 Å, R-factor ≤ 0.18) X-ray structures screened for nonredundancy and length (40–10,000 residues), as outlined in a previously published study.^25^ Additionally, we supplemented this data set with a set of 74 structures (*R* ≤ 2.5 Å) determined through neutron diffraction, as these structures are valuable for assigning the positions of hydrogen atoms (labeled through deuterium).

### Parameters for Aromatic–Aromatic Pairs

**Figure 1:**
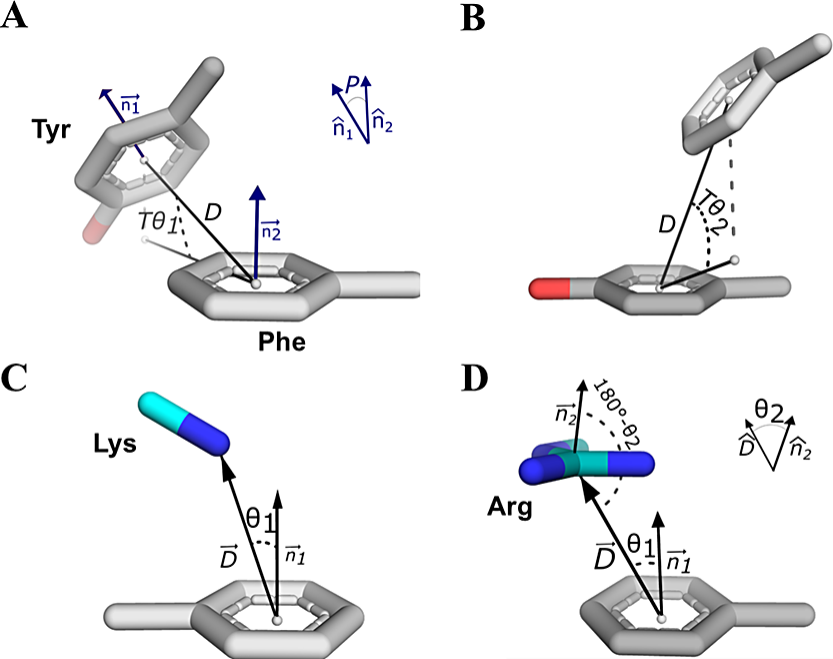
Selected geometric parameters to represent aromatic–aromatic and cationic–aromatic interactions. (A, B) Definition of parameters for an aromatic–aromatic pair, Phe–Tyr, where *D* is the distance between the centroids of the Phe and Tyr rings, *T*θ_1_ is the angle of elevation of the Tyr centroid relative to Phe, *T*θ_2_ is the angle of elevation of Phe relative to the Tyr centroid, and *P* is the angle between vectors normal to the two rings and yields *P* ≤ 90°. (C, D) Definition of parameters for cationic–aromatic amino acid pairs involving Lys, Arg, or Tyr. (C) Parameters for a Tyr–Lys pair, where θ_1_ describes the angle between the NZ atom of Lys and the normal of the Tyr ring’s plane (represented by the n_1_ vector), where *D* is the distance of the NZ atom from the ring centroid. (D) Parameters for the Tyr–Arg pair are as for panel (C), with the addition of parameter θ_2_, which denotes the angle between a vector normal to Arg’s plane and vector D.

We started our investigation by identifying pairs of aromatic amino acids. We applied a carbon–carbon approach previously defined for Phe–Phe pairs^27^ and broadened it to include aromatic amino acids Tyr and Trp. For each pairwise conformation, we calculated two independent variables: the distance between centroids (*D*) and the angle between aromatic ring planes (*P*). We further considered *T*θ_1_, the angle of elevation of the centroid of the ring of aromatic residue 1 relative to the plane of the ring of amino acid 2. Similarly, we determined *T*θ_2_, the angle of elevation of the centroid of the ring of aromatic residue 2 relative to the plane of the ring of amino acid 1. For example, the angle of elevation of the centroid of Tyr’s benzyl ring from the center of Phe’s ring is denoted as *T*θ_1_. Similarly, by projecting the center of Phe’s aromatic ring onto the plane of its Tyr partner ring, we calculated *T*θ_2_. For Trp residues, only the six-membered ring atoms were considered here. These parameters are further described in Figure 1A,B.

### Parameters for Cationic–Aromatic Pairs

We extended our analysis to pairs involving aromatic (Phe, Tyr, and Trp) and positively charged amino acids (Arg and Lys) that form cationic–aromatic pairs using the geometric parameters obtained from another work,^12^ as demonstrated in Figure 1C,D.

For each interacting pair in which Lys serves as the cationic member, we calculated the distance (*D*) between the N atom in the terminal zeta position of the Lys residue (the Lys NZ^32^ atom) and the centroid of the paired aromatic ring (Phe, Try, or Trp). We then calculated the angle between a vector connecting the NZ atom and the ring center to the normal vector of the ring’s plane (θ_1_) to calculate the angle of elevation of Lys compared with the plane of the partner ring. For cation–aromatic interactions involving Arg, the distance was measured from the center of the charge on the C atom in the terminal zeta position of the Arg residue (the Arg CZ atom). To describe the orientation of the Arg plane, an additional angle (θ_2_) was considered between the normal of the Arg plane and the direction of the CZ atom from the centroid (*D*). These pairs were then clustered according to their geometry (*D*, θ_1_, θ_2_) by employing the methodology described in the following section.

### Selected Pairs for Energetic Analysis

Using the geometrical parameters defined in Figure 1, we sought to pinpoint specific geometries to represent the conformational space occupied by the selected residue pairs. To that end, we conducted data clustering by applying the Gaussian mixture model,^33^ which clusters spatial data, in our case the PDB pairs, with an emphasis on the variance in the data. Thus, we allowed the clusters to adapt more abstract shapes (i.e., they were not limited to spherical shapes) than are allowed by more commonly used methods, such as K-means.^34^ The clustering algorithm was implemented using the scikit-learn library.^35^ For the smaller neutron-diffraction data set, we considered all defined pairs without clustering to ensure no important conformations were excluded.

### Binding Energies of Pairwise Interactions

Binding energies (being the difference in energy between the optimized pairwise conformation and the energies of the optimized separated residues) were then calculated for the representative pairs, selected based on clustering the sampled pairs, using ORCA 5.0.3^36^ software. For this purpose, each pair geometry was first briefly (<500 steps) optimized using the double-hybrid functional revDSD-PBE86-D4/QZ^37^ with the conductor-like polarizable continuum model (CPCM) as an implicit water solvent. The use of a polarizable implicit model within DFT-D calculations was previously shown to describe intermolecular interactions of biologically relevant molecules as accurately in water as in the gas phase.^38^ The CPCM model was previously used to calculate the p*K*_a_ of protonated and neutral (solvent-exposed) His residues and was found to provide an excellent balance between computational time and accuracy.^39^ The optimized geometries indicate a proximate local or global minimum energy conformation. Although our initial clustered points sampled the geometric space within the PDB, the optimized conformations might converge toward a distinct geometry. This convergence may indicate the influence of the protein’s environment on the isolated pairwise interactions. We note that while several minimal energy conformations obtained through QM optimizations were not present in our PDB structures data set, these conformations can be important for IDPs and are therefore included in our energetical analysis.

Some functionals that performed well on a benchmark aromatic–aromatic interaction data set (including H-bonds, π-stacks, and CH−π)^40^ did not perform as accurately on a data set of cation−π interactions.^30^ Thus, for the current study, we chose QM parameters that perform well on cation−π, π-stacking, and CH−π interactions.^30,40^ The reported binding energies were calculated using the diffused basis set def2-qzvppd (excluding hydrogen atoms) for revDSD-PBE86-D4, after a short validation for 20 pairs at the LNO-CCSD(T)^41^ level of calculations. For the latter, we applied the aug-cc-pV5Z basis set (cc-pV5Z for H atoms) using the MRCC program.^42^ These validations suggest that the binding energies deviate by 0.2 kcal/mol on average (∼3% error of the value) from the higher-level calculations.

### Geometrical Classification of Hydrogen-Bonded, CH−π, π-Stacking, and Cation−π Interactions

To compare the abundance and strengths of commonly discussed interactions, we categorized hydrogen-bonded pairs,^43^ CH−π pairs,^20^ π-stacking interactions,^44^ and cation−π pairs,^45^ as defined by geometric criteria established in previous research. These definitions rely on precise data concerning the positions of the hydrogen atoms and, consequently, we utilized only the QM-optimized pairwise configurations for these categorizations. We considered only geometries satisfying binding energies < −1 kcal/mol for any categorization of the four types of pairwise interactions considered, which is at least 5-fold greater than the average error for these calculations. We further note that the standard deviations for the average energies reported for each interaction might be overestimated, as they include geometries that span different conformational angular regions of our parameter space. Π-stacking interactions were identified based on the distance cutoff between the centroids of the aromatic rings and using angle definitions that define the orientation of the planes of the residues as approximately parallel. CH−π interactions are defined by the distance of the closest carbon (which donates the hydrogen) to the center of the π-acceptor system, along with the projected distance of the donated hydrogen on the π-system from this center. Finally, the identification of H-bonds includes determining the distance between the hydrogen donor and acceptor, the angle between the donor of the hydrogen atom and the acceptor atom, and the angle of the H-bond from the plane of the His ring. Additional details regarding these geometric classifications can be found in Supporting Information Figure S3.

## Results and Discussion

In this study, we aimed to quantify the energetic strengths and geometric prevalence of different interaction types involving aromatic amino acids under both hydrophilic and hydrophobic conditions within the protein, which represent solvent-exposed and -buried residues, respectively. While gas-phase energies are often considered to describe aromatic interactions in other studies, solvent-exposed energies may correspond to their contributions to disordered regions or IDPs.

To ensure the accuracy of the calculated energies, we opted for QM calculations known for their precision in determining the energies of selected noncovalent pairwise interactions sampled from high-resolution crystal structures. These energies were then mapped onto the population density of the geometries of these pairwise interactions in the high-resolution structured protein data set. Such mapping enables the abundance of pairwise interactions in a certain conformational space to be correlated to their binding energies. Employing this approach, we compared two distinct types of interactions: aromatic–aromatic pairs (Figure 1A,B) and cationic–aromatic pairs (Figure 1C,D). The former group encompasses Phe–Phe, Phe–Tyr, Phe–Trp, Tyr–Tyr, Trp–Tyr, and Trp–Trp pairs, whereas the latter includes Lys–Phe, Lys–Tyr, Lys–Trp, Arg–Phe, Arg–Tyr, and Arg–Trp pairs.

### π–π Interactions Are Stable but Less Prevalent in Structured Proteins

π–π interactions are often perceived as key stabilizers of protein structures and thus their energetic contributions are commonly discussed in studies of structured and disordered protein conformations.^10,25,29,40,46,47^ Following a debate regarding the accuracy of this perception,^23^ we sought to re-evaluate their energies and compare them with the energetic contributions of CH−π, cation−π, and H-bonding interactions using QM calculations (see Methods), as well as to evaluate the abundance of π–π interactions in structured proteins. Our QM calculations for the strength of π–π interactions (see Table 1) were performed for both solvated and gas-phase conditions. We observed that these interactions become more attractive with increasing π-system size, in both aqueous solvents and the gas phase. For example, in water, binding energies strengthen with increasing π-system size from an average of −3.3 kcal/mol for Phe–Phe pairs to −4.2 kcal/mol for Phe–Trp and reach −5.2 kcal/mol for Trp–Trp pairs.

**Table 1:** Average Binding Energies (kcal/mol) of Pairwise Aromatic–Aromatic Interactions^a^

	CH−π^b^		π-stacked^b^		H-bonds^b^	
	water	gas-phase	water	gas-phase	water	gas-phase
Phe–Phe	–2.9 ± 0.3	–3.4 ± 0.4	–3.3 ± 0.3	–3.7 ± 0.5		
Phe–Tyr	–3.1 ± 0.3	–3.7 ± 0.5	–3.5 ± 0.3	–4.0 ± 0.7		
Phe–Trp	–3.4 ± 0.6	–4.2 ± 0.9	–4.2 ± 0.5	–4.7 ± 0.5		
Tyr–Tyr	–3.4 ± 0.4	–4.1 ± 0.8	–3.7 ± 0.3	–4.3 ± 0.6	–4.8 ± 0.3	–6.5 ± 0.4
Tyr–Trp	–3.7 ± 0.6	–4.5 ± 1.1	–4.4 ± 0.6	–5.0 ± 0.8	–4.4 ± 0.5	–6.6 ± 0.4
Trp–Trp	–4.2 ± 0.6	–5.0 ± 1.1	–5.2 ± 0.43	–5.3 ± 0.7		

a Considering only interactions with binding energies below a threshold of −1 kcal/mol.

b CH−π, π-stacked, and H-bonding pairwise interactions were classified based on geometric parameters, as described in Methods.

Interestingly, in a solvent environment, π-stacked interactions between Trp–Tyr pairs displayed an average binding energy of −4.4 kcal/mol, which is only slightly (∼12%) weaker than the gas-phase value (−5.0 kcal/mol). Hence, we observe that π-stacked interactions not only approach the strength of the inarguably strong and abundant H-bonds under solvent conditions (−4.4 kcal/mol for Tyr–Trp pairs) but also exhibit less sensitivity to the presence of a solvent when compared with the ∼2 kcal/mol gap observed in H-bonds (which are ∼30% weaker in solvents). Overall, our calculations suggest that π-stacked interactions possess the strongest binding energies among all interactions that aromatic–aromatic pairs can participate in, which justifies the significant role attributed to them in governing protein stability.

**Figure 2:**
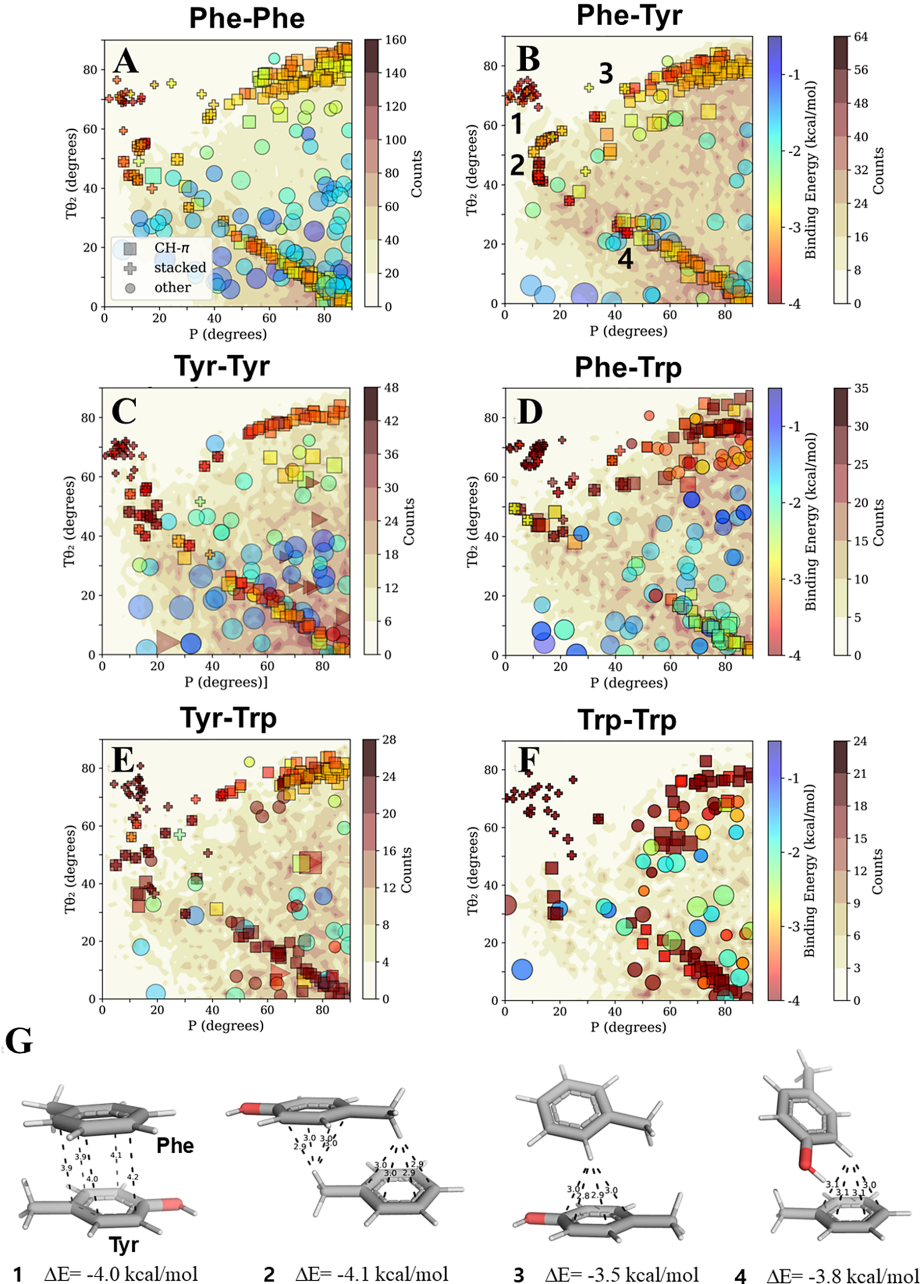
π–π interactions between Phe, Tyr, and Trp. Binding energies (rainbow colorbar) from QM calculations of pairwise interactions in aqueous solution between Phe–Phe (A), Phe–Tyr (B), Tyr–Tyr (C), Phe–Trp (D), Tyr–Trp (E), and Trp–Trp (F) pairs projected onto density contour gradients (white–brown colorbar) created by counting the frequency of each pair geometry as sampled from 6535 high-resolution PDB structures. The geometries of pairwise interactions are mapped in terms of angular parameters *P* and *T*θ_2_, these being two of the four geometric measures required to represent all pairwise interactions (see Figure 1). For the symmetric cases (i.e., Phe–Phe, Tyr–Tyr, and Trp–Trp), *T*θ_2_ density values also include *T*θ_1_ to account for randomly choosing one residue to calculate *T*θ_1_ rather than the other. The quantum calculations were performed on selected pairs from those found in the sampled database. We note that each selected pair underwent energetic optimization, and consequently, its final geometry may deviate from its original starting structure. The pairwise interactions are categorized as π-stacked or CH−π based on distance and angle cutoffs (see Methods for further details). All other geometries are classified as “other”. The size of the symbol represents the *D* geometric parameter (i.e., the distance *D* between the centroids of the two aromatic rings). (G) Selected pairwise geometries and their corresponding binding energies for Phe–Tyr pairwise interactions (geometries **1–4**; see panel B). These pairwise interactions are minimal energy geometries of Phe–Tyr characterized by CH−π or π–π interactions. Interaction-type categorization follows Figure S1.

However, while our QM calculations support the stabilizing potential of π-stacked interactions, this need not necessarily correlate with their prevalence in experimentally resolved protein structures. With this in mind, we mapped the calculated QM binding energies for selected pairwise interactions onto the contour representing the relative populations of the pairs in protein structures. The pairs were classified according to specific geometric parameters such as the angle, *P*, between the normal of the two aromatic rings and the elevation, *T*θ_2_, of one ring relative to the centroid of its paired ring (Figure 1A,B). These maps are shown in Figure 2 for Phe–Phe, Phe–Tyr, Phe–Trp, Tyr–Tyr, Tyr–Trp, and Trp–Trp pairs in aqueous solvents, whereas gas-phase maps for these pairs can be found in Supporting Figure S4. On the basis of its geometry, each pairwise interaction was classified as a π-stacked, CH−π, or H-bonding interaction, as appropriate.

We observed that the most energetically favorable Phe–Phe π-stacked interactions (Figure 2A) are restricted to a specific angular parameter space. These interactions can either exist independently in the space defined by 60° < *T*θ_2_ < 80°, 0° < *P* < 20° or overlap with CH−π interactions in the space delineated by 40° < *T*θ_2_ < 60°, 0° < *P* < 30°. This holds also for other pairs, such as Tyr–Tyr, Phe–Trp, Phe–Tyr, and Tyr–Trp (Figure 2). With respect to Phe–Phe interactions, this observation is in line with previous work^48^ demonstrating that the lowest energy structure for toluene dimers (for the structure, see Supporting Figure S1) corresponds to a stacked conformation. However, it is crucial to note that the method used by the prior study was subsequently found to deviate significantly from actual π–π stacking energies, highlighting the need for careful evaluation.^49^ It is also worth noting that the common practice of representing Phe using a symmetric benzene ring, rather than a toluene group, can mislead.^2,50,51^ While the global minimum for the toluene dimer is stacked, the benzene dimer exhibits perpendicular minimum energy conformations in preference to parallel ones.^52^ This distinction is critical, as the symmetric benzene representation overlooks the electron delocalization effects present in the actual Phe side chain.

Surprisingly, despite calculations indicating strong π-stacking interactions, we observed that for Phe–Phe, where *T*θ_2_ > 65°, *P* < 30°, purely π-stacked interactions are rarely found in high-resolution PDB data sets. In contrast, we identified a minor, yet notable, population of π-stacked interactions overlapping with the CH−π contributions (40 ° < *T*θ_2_ < 60°, 5° < *P* < 30°), which aligns with the reported global minimum structure encompassing antiparallel methyl groups.^48^ We note that previously, a high abundance of π-stacked geometries was reported for Phe–Phe interactions in proteins,^9,53^ which can be seen in our data if a lower cutoff distance of *D* ≤ 5 Å is used (refer to Supporting Information Figure S5A,B). However, such a cutoff excludes several conformations of perpendicular or tilted orientations that Phe–Phe pairs can take. For Phe–Phe, it was also reported that the conformations are sampled as if by chance,^27^ which is supported by our analysis when a cutoff of *D* > 5 Å is used (see Supporting Information Figure S5C,D).

This apparent discrepancy between the notable enthalpic contribution of π-stacked interactions and their low abundance in proteins is likely a function of the environment in which the protein residue pairs interact, which tends to be more hydrophobic than solvent-exposed, as further discussed in Supporting Information Section S4. The existence of a solvent effect is further supported by the positive correlation observed between the frequency of π-stacked conformations and the number of water molecules in the vicinity of the interacting pair,^25^ with π-stacked interactions found to be much more frequent in loop and turn protein regions (which are solvent-exposed) compared with internal protein regions (which are not). These observations suggest that for disordered proteins, π-stacked interactions may be not only energetically attractive but also much more common as a key stabilizer.

Among all the aromatic residue pairs examined, both the homogeneous pairs (Phe–Phe, Tyr–Tyr, and Trp–Trp) and the heterogeneous pairs (Phe–Tyr, Phe–Trp, and Tyr–Trp), we observed π-stacking to be a low-abundance conformation in structured proteins. In this context, it is particularly interesting to compare the maps of Phe–Phe and Phe–Tyr, where the introduction of only a single hydroxyl group to Phe–Tyr pairs breaks the symmetry observed in Phe–Phe pairs and influences these interactions. Indeed, there is a minor population of π-stacked Phe–Tyr pairs (Figure 2B), whereas this region is largely unpopulated by π-stacked Phe–Phe pairs (Figure 2A). The presence of π-stacked populations is also observed for Tyr–Tyr (Figure 2C) and Tyr–Trp (Figure 2E) pairs but not for Trp–Trp or Phe–Trp pairs, thus suggesting that the effect of the hydroxyl group is not related to directionality introduced by breaking symmetry in Phe–Tyr pairs but rather is an intrinsic ability of the Tyr residue. It is likely that the ability of the Tyr residue to participate in H-bonds (with either backbone or side chain atoms) may drive Tyr to adopt a specific orientation that both extends its geometrical 2D space and diverges from randomly expected distributions.

### CH−π Interactions Are Weak but Ubiquitous and Support Other Interactions

Following our observation of the low population of π-stacked geometries of Phe–Phe pairs in structured proteins, where some of the most stable interactions are further stabilized by CH−π contacts, we aimed to understand the properties of CH−π interactions and their abundance. We note that, in this work, we treat the previously studied^54^ perpendicular T-shaped “π–π interactions” as falling within the definition of “CH−π interactions”, as supported by several studies.^28,55−57^

**Figure 3:**
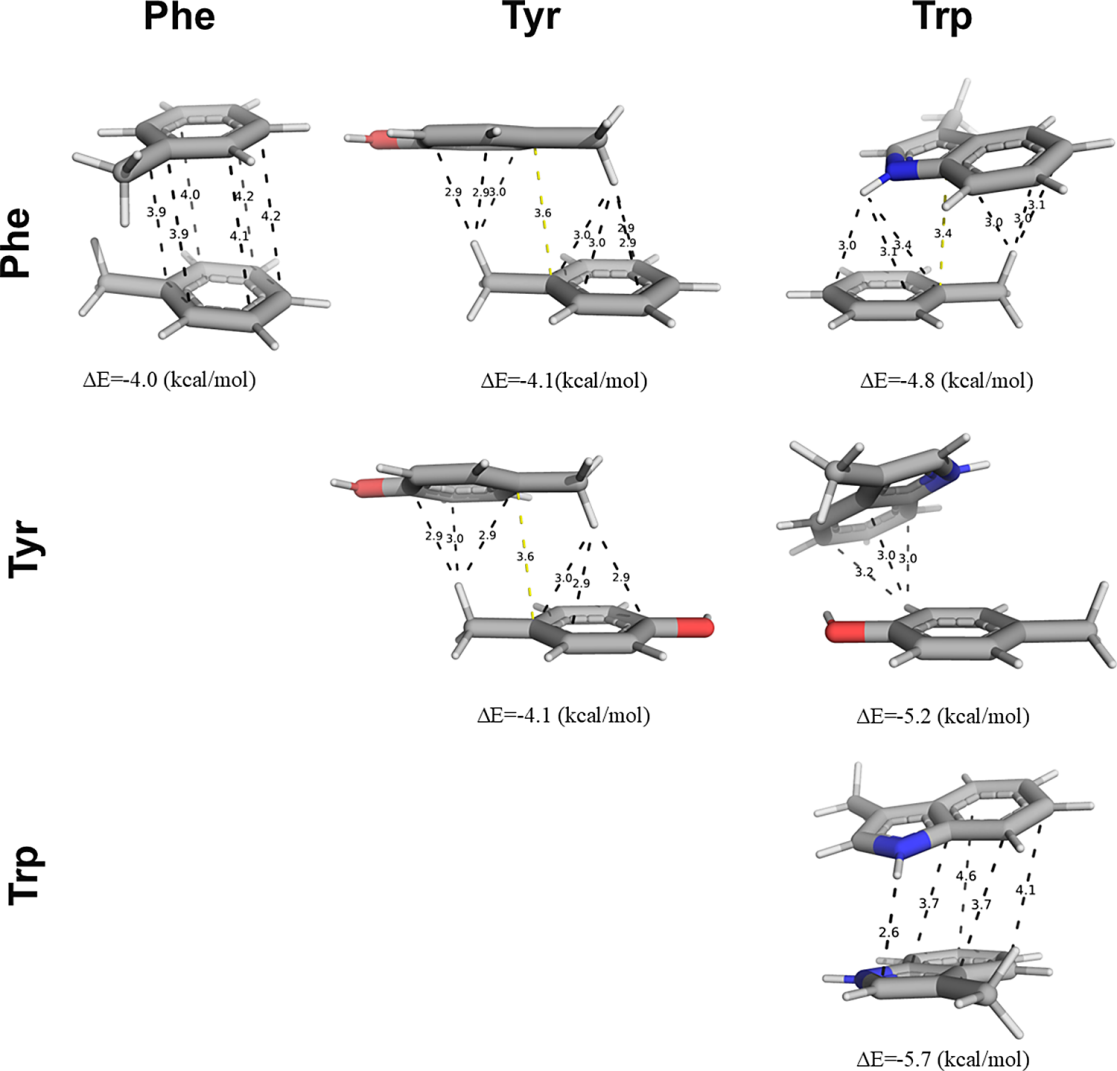
Aromatic–aromatic pairs interacting through π-stacking. A configuration for each pairwise interaction between Phe, Tyr, and Trp is shown together with its corresponding binding energy, thereby illustrating the relationship between π–π and CH−π interactions along with their minimal binding energy geometries, categorized according to Figure S1.

Interestingly, in the lowest energy Phe–Tyr stacked conformation, we observed the simultaneous occurrence of two CH−π interactions, as illustrated in Figure 3. The remarkable similarity in the stacked global minimum geometry and energies (−4.1 kcal/mol) of Phe–Tyr and Tyr–Tyr (Figure 3) suggests that the major contributor to the stacked conformation adopted is not the dipole of Tyr but rather the combined effect of multiple CH−π interactions. This highlights a potential limitation in the current definition of stacked interactions, which may not fully capture the complexity introduced by contributions from the overlapping CH−π interactions. Consequently, incorporating energetic considerations alongside geometrical mapping is crucial for obtaining a more comprehensive understanding. The recurring presence of CH−π interactions in the minimal energy stacked conformations of Phe–Tyr, Phe–Trp, Tyr–Tyr, and Tyr–Trp pairs (Figure 3) emphasizes the necessity to consider these additional low-energy CH−π interactions within the overall interaction system. Considering CH−π as standalone interactions, we observed that they appear in the most populated regions for any aromatic–aromatic pair (Figure 2) where *T*θ_2_ < 30°, *P* > 70°. However, this region is expected to be most populated by normally distributed orientations of planes and may encompass several conformations (Table 2 in ref (27)) so it cannot be concluded that this density originates solely from standalone CH−π interactions. This observation, coupled with the remarkable occurrence reported^5,20^ for these poorly discussed CH−π interactions, suggests the need for further inspection of the geometrical restrictions and strengths of CH−π also as standalone interactions.

We compared CH−π interactions with other commonly discussed interaction types. Unlike the highly directional nature of hydrogen bonds^58^ and the narrow 2D space occupied by stacked geometries (Figure 2), we found that CH−π interactions exhibit a broader distribution across various plane angles. This suggests a lower specificity, with more degrees of freedom, for CH−π compared to π-stacked interactions. Additionally, the reported cation−π interactions (see the later section) exhibit a high specificity following the detection of a sharp θ_1_ angle distribution, unlike the broad distribution seen in the CH−π scenario.^59^

We thus found it interesting to examine whether the weak specificity of CH−π interactions, which is unique among all other discussed interaction types, necessarily correlates with their strength. It is expected that the strength of “pure” π-stacked interactions should increase with the size of the π-system,^56^ whereas the strength of CH−π interactions should increase with the acidity of the C-H donor group.^57^ As shown in Table 1, the average strength of solvated CH−π interactions increases from −2.9 kcal/mol for Phe–Phe pairs to −3.4 kcal/mol for Phe–Trp pairs and culminates at −4.2 kcal/mol for Trp–Trp pairs, a trend that is more moderate compared with that observed for π-stacked interactions. In fact, we found that the strength of CH−π interactions is indeed very weakly dependent on the identity of the donor residue (see the discussion in Supporting Information Section S6). Finally, considering solvent effects, we observed that aromatic pairs engaging in CH−π interactions lose only around ∼0.5 kcal/mol of their attractive binding energy (see Table 2) when solvated compared with gas-phase calculations, with the exception of Trp (refer to Section S6). Indeed, it was previously shown that CH−π interactions have a large dispersion contribution, which shields solvent effects and thus conserves the strength of these interactions in both polar and nonpolar environments.^60^

**Table 2:** Average Binding Energies (kcal/mol) of Pairwise Cationic–Aromatic Interactions^a^

	CH−π^b^		cation−π^b^		H-bonds^b^	
	water	gas-phase	water	gas-phase	water	gas-phase
Phe–Lys	–2.2 ± 0.2	–12 ± 1.6	–2.4 ± 0.4	–12 ± 3.5		
Tyr–Lys	–2.3 ± 0.2	–12 ± 1.6	–2.3 ± 0.4	–12 ± 2.7	–3.9 ± 0.5	–18 ± 0.6
Trp–Lys	–3.0 ± 0.7	–15 ± 3.7	–3.1 ± 0.8	–16 ± 4.9		
Phe–Arg	–3.2 ± 0.4	–8.8 ± 0.8	–3.4 ± 0.4	–10 ± 1.5		
Tyr–Arg	–3.4 ± 0.4	–9.6 ± 1.2	–3.7 ± 0.4	–11 ± 1.3	–4.0 ± 0.3	–13 ± 1.5
Trp–Arg	–4.4 ± 0.6	–12 ± 1.2	–4.7 ± 0.6	–15 ± 2.3		

a Considering only interactions with binding energies below a threshold of −1 kcal/mol.

b CH−π, cation−π, and H-bonding of pairwise interactions were classified based on geometric parameters, as described in Methods.

Overall, we observed that the strength of these “pure” CH−π interactions is not predominantly dependent on solvent or geometry (i.e., on *P* or *T*θ_2_, see Figure 2A,B) and is only weakly dependent on the identity of the donor or acceptor, as demonstrated in Table S2 where the donors Phe (in Phe–Tyr pairs), Tyr (in Phe–Tyr pairs), and Trp (in Phe–Trp pairs) participate in CH−π interacting pairs with a binding energy of around −3 kcal/mol. This characteristic ultimately facilitates the high observed prevalence of these interactions in proteins.^20^

### Cation−π Interactions Involving Arg Are Stronger and More Prevalent than Those Involving Lys

Recent studies highlighted the prevalence of cation−π interactions in proteins,^12,16,18,61^ along with efforts to evaluate accurately their energies^30,50,62^ and, thus, also their role in stabilizing protein structures. We limit the discussion of their strength to solvated pairs, as it is expected that a cationic residue in a protein will be rather solvent-exposed, and since cation−π interactions are of particular interest as stabilizing forces in IDPs.^7,8,25^ It has been shown that the strength of these interactions increases with the size of the aromatic ring.^63^ Indeed, based on our calculations, we observed that the average binding energies (Table 2) in solvated regions correlate with the size of the aromatic system. When Arg serves as the cation, the energies range from −3.4 kcal/mol for Arg–Phe pairs to −4.7 kcal/mol for Arg–Trp pairs. For cationic Lys, we observed much weaker average binding energies of −2.4 kcal/mol for Lys–Phe and Lys–Tyr and −3.2 kcal/mol for Lys–Trp pairs. Thus, Arg is a more attractive cation, possibly due to coupled contributions from dispersion interactions through its additional π-system, as reported in a recent work, highlighting the higher affinity for Arg as a cation compared to Lys with aromatic residues in IDPs.^64^ This apparent difference between the affinity of Arg and Lys cations is not evident when the atoms are simply considering H-bonds. We found that Lys–Tyr H-bonds contribute −3.9 kcal/mol to the average binding energies, which approaches the contribution from Arg–Tyr H-bonds at −4.0 kcal/mol. Thus, on average, H-bond strengths are not dependent on the identity of the donor (Arg/Lys), in contrast to the cation−π case. These observations are even more intriguing because Arg–Tyr pairs are found to be the most prevalent among cation–aromatic pairs,^2^ despite the natural frequency of Lys (∼6%) being higher than that of Arg (∼5%) in our structured PDB data set, thus suggesting a possibly important contribution from cation−π interactions among these pairs.

**Figure 4:**
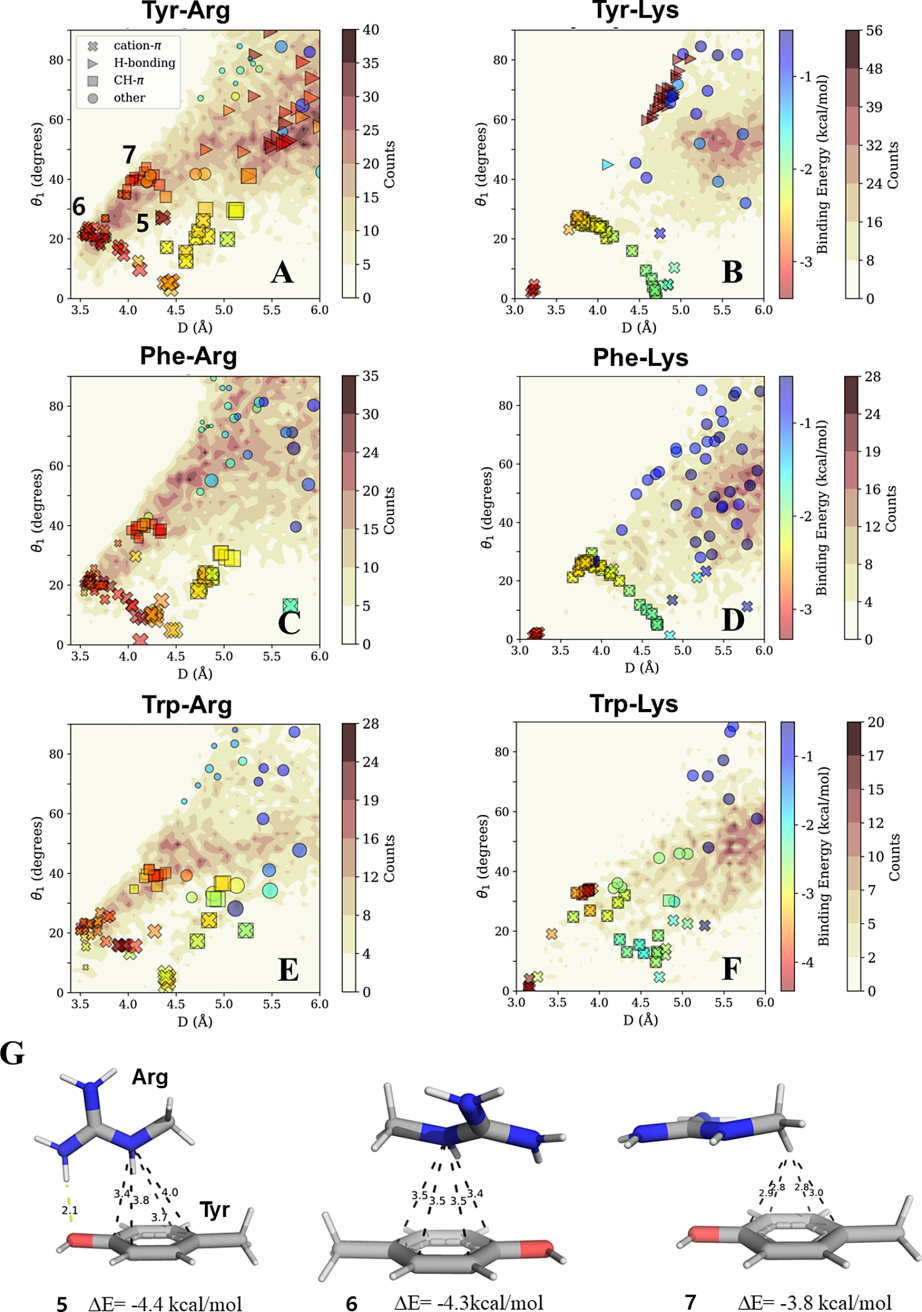
Cation−π interactions between Phe, Tyr, or Trp and Arg or Lys. The binding energies (rainbow colorbar) of pairwise interactions in aqueous solvent were calculated for pairs selected from geometries obtained from high-resolution protein structures and projected onto density contour maps (white–brown colorbar) calculated as described in Figure 2. The cation−π interactions are shown for interactions between (A, B) Tyr, (C, D) Phe, or (E, F) Trp π-systems and (left column) an Arg or (right column) Lys cation. The pairwise interactions are categorized geometrically as H-bonding, CH−π, π-stacked, and other. (G) Three interacting Tyr–Arg pairs possess H-bonding, π-stacked and CH−π geometries (geometries **5–7**).

**Figure 5:**
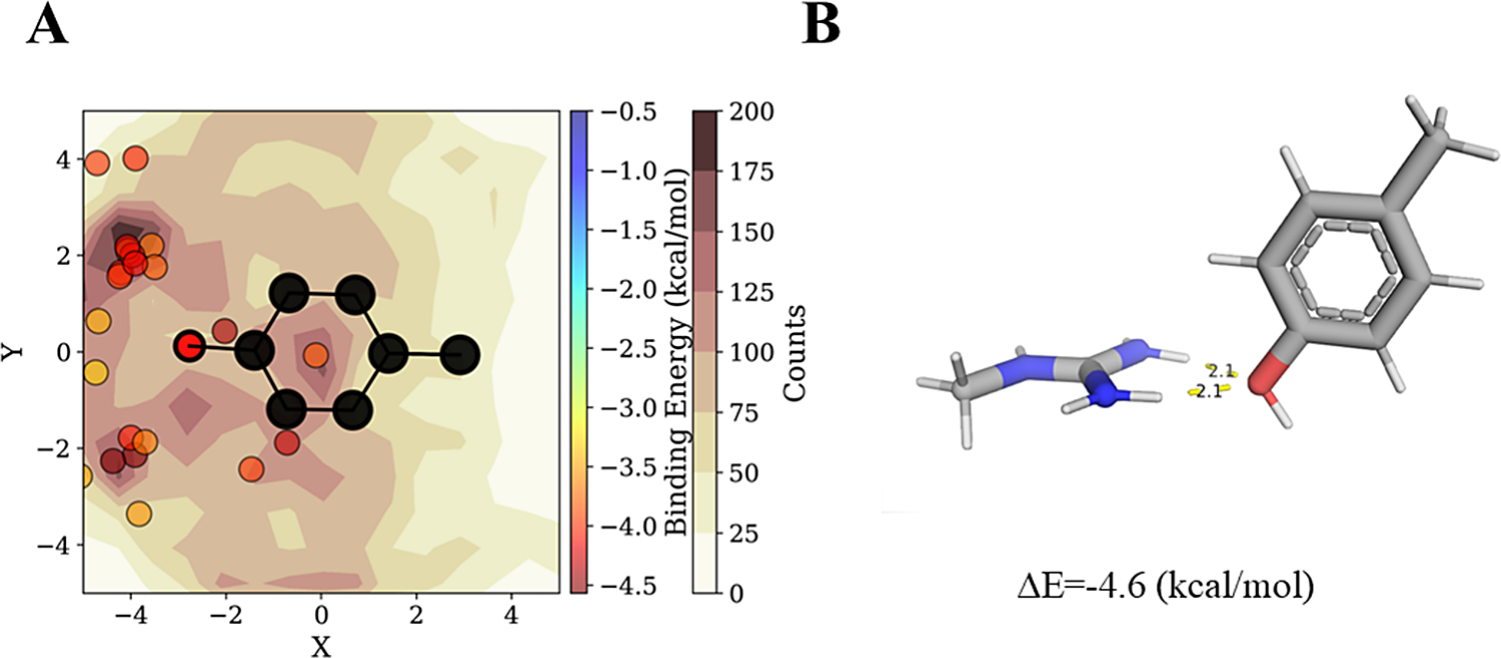
Geometry of Arg–Tyr pairs engaged in hydrogen bonding. (A) Mapping of the NH1 atom of Arg relative to Tyr, where the circles represent configurations identified as hydrogen-bonded interacting pairs. The QM energy of these pairs is in agreement with those of the most populated geometries found in the PDB. (B) Lowest energy conformation of Tyr–Arg pairs was identified as H-bonded.

To understand whether the energies of these attractive cation−π interactions correlate with their abundance, we mapped them as an overlay onto their observed PDB prevalence in the corresponding geometric space. The discussed interaction maps of Arg or Lys with Phe, Tyr, or Trp in aqueous solvents are provided in Figure 4, whereas gas-phase maps of Arg/Lys-Tyr can be found in Supporting Figure S7. Consistent with another study that used partial charges and simple electrostatic and van der Waals calculations,^65^ we observed that cation−π interactions are indeed confined to angles of θ_1_ < 40° (see Figure 4A–F). Arg–Phe/Tyr/Trp solvated pairs preferably occupy stacked geometries (in which the Arg plane is nearly parallel to the aromatic ring plane), with these characterized by minimal distances (*D* ∼ 3.5 Å), low θ_1_ (∼20°), and small θ_2_ values (see Figure 4G conformation 6). Their high observed prevalence is intriguing, considering the expectation of interactions increasing in likelihood at greater distances because greater *D* values bring more potential partner residues into the contact range. This expected large-distance bias is indeed observed in the high population of energetically less favorable interactions for Tyr–Lys, Phe–Lys, and Trp–Lys (Figure 4B,D,F). However, we note an exception for Tyr–Arg pairs (Figure 4A) when compared with density maps of Phe–Arg (Figure 4C) or Trp–Arg (Figure 4E), as only Tyr can participate in H-bonds among these pairs. Indeed, for Tyr–Arg, the densely populated region in geometries where θ_1_ > 45° is populated by very attractive H-bonds. This preference is also supported by a similar analysis of the projection of the Arg NH1 atom around the aromatic ring of Tyr, as illustrated in Figure 5A. In contrast to Lys, Arg can simultaneously participate in multiple hydrogen bonds with Tyr oxygen as shown in Figure 5B. Additionally, compared to Lys, Arg is more frequently found in a charged state, even when placed inside hydrophobic cores,^66^ in which the strengths of H-bonds are much more favorable compared with solvated conditions (see Table 2). These properties facilitate the prevalence of Tyr–Arg as a key stabilizer, even in the hydrophobic environment of the interior of structured proteins.

Intriguingly cationic–aromatic pairs involving Lys demonstrate different behavior compared with those involving Arg. The prevalence of cation−π geometries in Arg-containing pairs (Figure 4A,C,E) is not evident in those involving Lys, for which cation−π geometries appear to be infrequently populated (Figure 4B,D,F). This is consistent with previous studies reporting that Arg–aromatic interactions are much more commonly observed than Lys–aromatic interactions.^19,28^ This difference can be attributed to the considerably lower strengths of non-H-bonding aqueous interactions involving Lys (∼ −2.4 kcal/mol) compared with Arg (∼ −3.4 to −3.7 kcal/mol) (Table 2).

**Figure 6:**
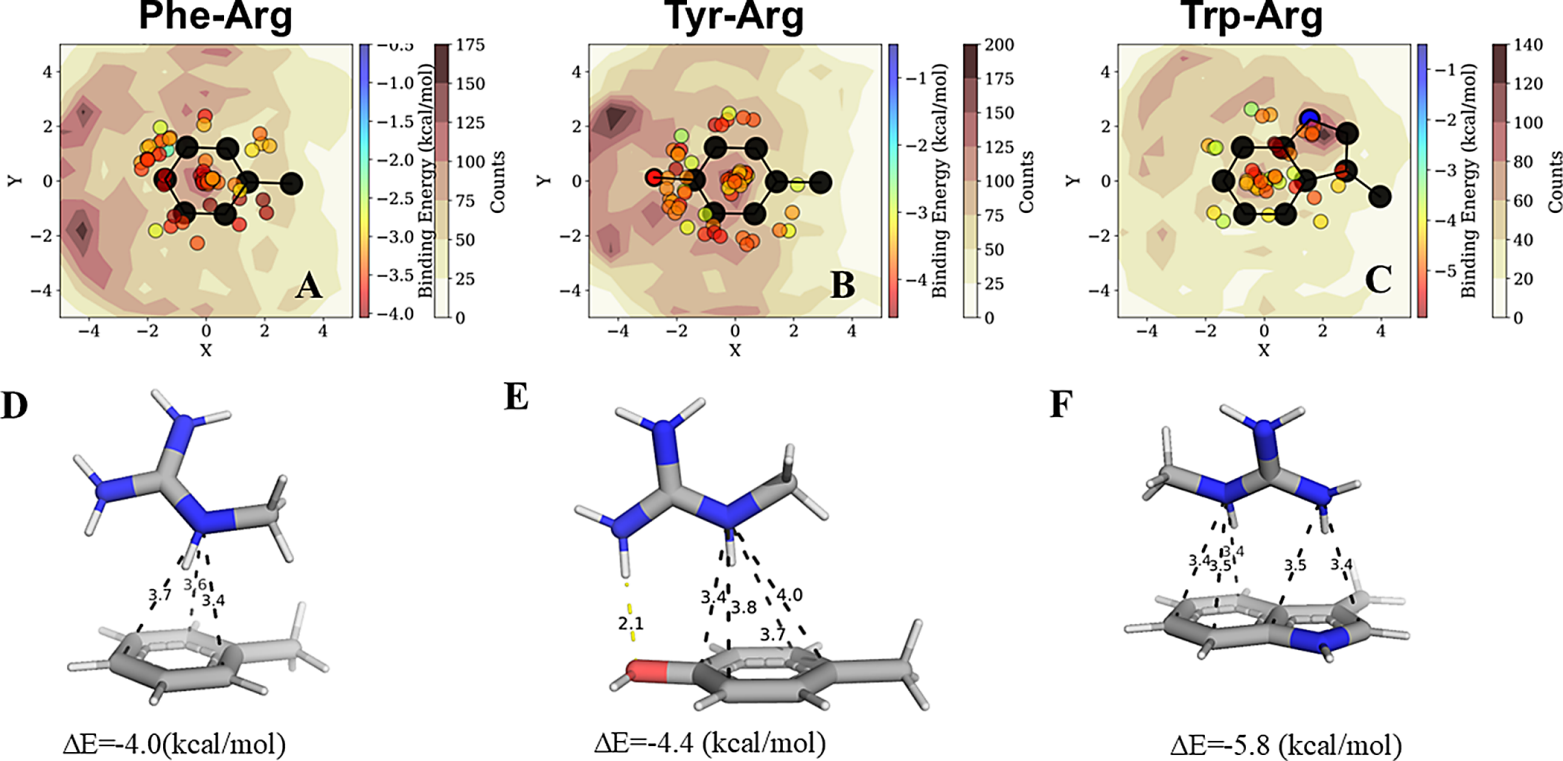
Geometries of cation−π interactions involving Arg cations with Phe, Tyr, and Trp π-systems. (A–C) Maps of the position of the NH1 atom of Arg (shown by circles) relative to (A) Phe, (B) Tyr, and (C) Trp π-systems. The circles correspond to selected pairwise geometries, categorized as cation−π, whose binding energies (rainbow colorbar) were calculated using QM and whose population is shown on the contour map (white–brown colorbar). (D–F) Conformations with the lowest energy are for the Phe–Arg, Tyr–Arg, and Trp–Arg cation−π pairs, respectively.

With this understanding, we further investigated the nature of cation−π interactions between Arg and various aromatic residues. We observed that the Arg NH1 atom frequently interacts with the centroids of Phe, Tyr, and Trp in cation−π interactions (Figure 6A–C). Notably, the NH1 atom occupies a position above both Trp rings. The interactions of Arg with the Phe and Tyr π-systems produce strikingly similar density count maps (Figure 6A,B), including high-density areas at low *X* values of *X* < −3, despite the inability of Phe to participate in H-bonds akin to those of Tyr with the Arg NH1 group. The high-density areas may be a result of other interaction types possessing a variety of geometries or could simply indicate that these configurations are statistically probable. The latter can arise from the distance cutoff analysis in that more distant configurations are more likely to be occupied, even by chance.

The minimal energy geometries presented in Figure 6D–F showcase a favorable T-shape-like geometry for these aromatic interactions, where the Arg NE atom lies closest to the centroids of the 6-membered rings. This is surprising considering the very low observed occurrence of NE in proximity to the Phe ring centroid (Figure S8B). This observation is even more surprising, given the previously identified preference of Arg–Tyr pairs to adopt a stacked conformation over a T-shaped orientation. The observations for Tyr–Arg and Trp–Arg interactions hint at additional stabilities potentially arising from H-bonding or further cation−π interactions. For Phe, however, this is an unexpected observation, because of the absence of additional contributions to these interactions. It appears that for Phe, the strengths of stacked cation−π conformations are not highly dependent on the elevation angle or distance (see Figure 4C). This reinforces a recurring pattern of Phe participating in interactions with geometries that might be sampled by chance rather than by specific energetic preferences. Based on our observations, we conclude that the common use of a single minimal energy geometry to infer the contributions to binding energy attributable to cation−π interactions^47,62^ may be misleading, and it is therefore necessary to study the energies of additional sampled geometries and consider their prevalence in proteins.

Following our observation of densely populated CH−π interaction regions for aromatic–aromatic pairs in structured proteins, we turned to investigating the population of CH−π interactions in cationic–aromatic pairs. The interaction maps for Tyr–Arg (Figure 4A) indeed show that some cation−π interactions overlap with CH−π interactions (*D* ∼ 4 Å, 35° < θ_1_ < 40°), suggesting coupled contributions. These coupled contributions are particularly interesting in the context of interactions involving Lys, where the majority of cation−π interactions appear to be defined also as CH−π interactions (see Figure 4B,D,F), yet are infrequently populated. The observation that few interactions deviate from the calculated conformations may be a result of CH−π interactions involving aliphatic carbons that were truncated from the Lys side chain and that are excluded in the QM calculations (see Supporting Information Section S9).

### Relative Binding Energies of Pairwise Interactions in Proteins and Potential Synergism between Various Binding Interactions

**Figure 7:**
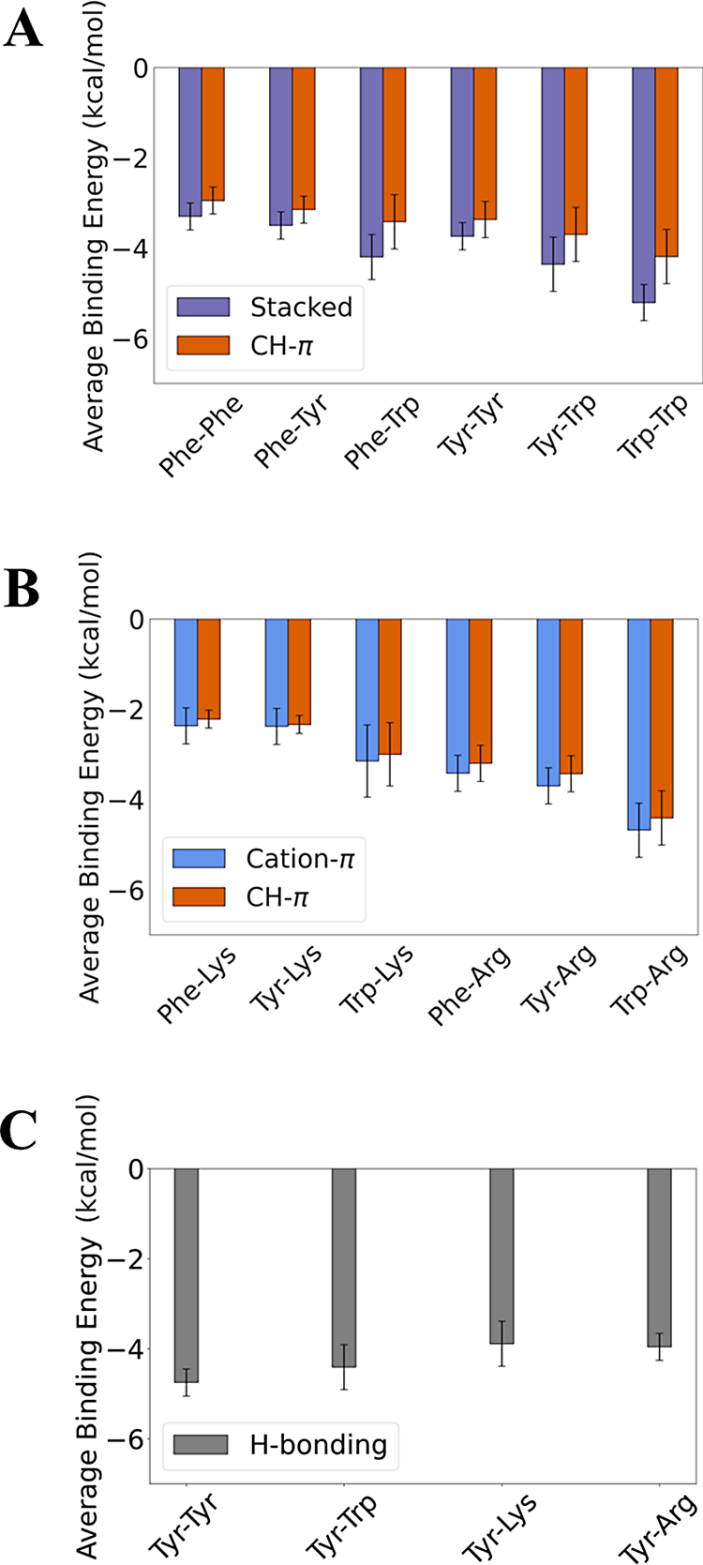
Summary of binding energies for pairwise interactions involving Phe, Tyr, Trp, Arg, and Lys residues. Average binding energies of pairwise interactions are categorized into three groups (based on geometric parameters): (A) π-stacked (purple) and CH−π (red) motifs involve solely aromatic residues Phe, Tyr, and Trp. (B) Cation−π (blue) and CH−π (red) interactions occur between the π electrons of a Phe, Tyr, or Trp residue and a cation or CH group from a basic Lys or Arg residue. (C) H-bonding interactions between Tyr and a Tyr, Trp, Lys, or Arg residue.

A summary of all of the QM binding energies for pairwise interactions between Phe, Tyr, Trp, Lys, and Arg as classified on the basis of the nature of their interactions is presented in Figure 7. It is evident that similar binding energies can be achieved via π–π, cation−π, CH−π, and hydrogen-bonding interactions, yet the strength of the binding energy in each case is highly dependent on the particular pair of residues involved (Figure 7). Formation of π–π interactions can contribute more than 3 kcal/mol to the binding energy, which is similar to the contribution from hydrogen bonding. CH−π pairs provide a slightly lower energy than do π–π interactions, yet their contribution is sufficiently significant to highlight their potential to contribute to protein stability and tune the local structure. Cation−π interactions, especially those involving Arg rather than Lys, approach the strength of aromatic–aromatic π-stacked interactions and even H-bonds. The abundance of both cation−π and CH−π interactions correlates with these energies, supporting the important contributions of those interactions to structured proteins, as observed from contact frequency per residue analysis of the neutron-diffraction data set (see SI Figure S10). These insights may be also valid to data sets with lower resolutions as in many instances the QM-calculated binding energies are similar within small deviations of angular angles, as demonstrated in the clusters represented by conformations 1, 2, and 3 in Figure 2G and conformations 5, 6, and 7 in Figure 4G.

**Figure 8:**
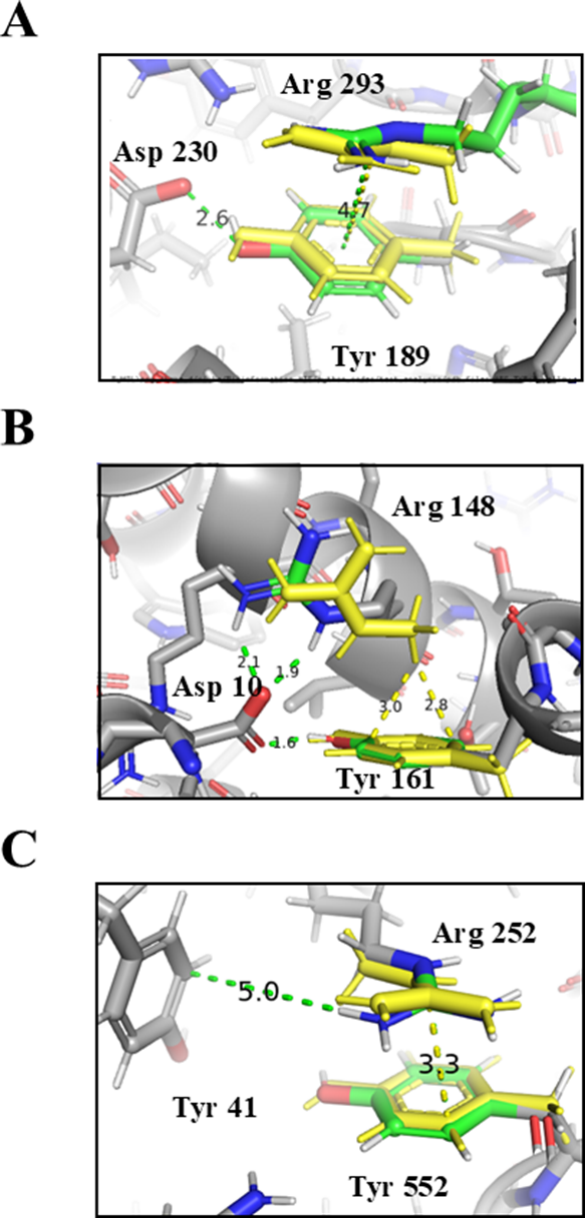
Synergism between pairwise interactions in proteins. QM calculations of pairwise interactions may experience some structural deviation when modeled in isolation compared with modeling in the context of the whole protein. Three examples are shown for pairwise calculations. (A) Pairwise interactions between Tyr189 and Arg293 (colored yellow) extracted from PDB ID 2R24 chain A. The yellow dashed line represents the cation−π interaction, identified by PyMOL, for the QM optimized pair with a distance of 4.7 Å. The calculated QM binding energy of this interaction is −4.3 kcal/mol. (B) Pairwise interactions between Tyr23 and Arg19 extracted from PDB ID 5ZN0 chain A, where the yellow dashed lines represent the CH−π interaction. The calculated QM binding energy of this interaction is −2.8 kcal/mol. (C) Pairwise interactions between Arg252 and Tyr552 extracted from PDB ID 7WNO chain X, where the yellow dashed line represents the simultaneous cation−π interactions of the original structure. The calculated QM binding energy of this interaction is −4.0 kcal/mol.

Our analysis of pairwise interactions extracted from high-resolution protein structures was rooted in the assumption that if the contributions of these interactions are energetically vital, then the conformations should remain similar when taken out of the protein context. However, the significance of these interactions should also be considered within the context of the structured protein, as shown in Figure 8. For example, Figure 8A shows that removing the protein structure context from a Tyr–Arg pair that bonds via a cation−π interaction does not significantly alter the QM-predicted low-energy geometry (yellow in Figure 8A compared with green), despite the exclusion of the hydrogen bond to Asp. This minor structural deviation from the optimal geometry in the context of the protein is expected, given the strong −4.3 kcal/mol contribution from the cation−π interaction in this geometry, and is further supported by the previously discussed weak distance dependence of cation−π interaction energies.^67^ By contrast, ignoring a hydrogen bond between Tyr and Asp, as well as a salt bridge between Arg and the same Asp residue, as shown in Figure 8B, results in a QM-calculated geometry stabilized via CH−π interactions, which diverges from the initial structure and the nature of the initial interaction. In this case, the calculated binding energy contributed merely −2.8 kcal/mol. Finally, when multiple aromatic residues lie close to an Arg residue, each interaction needs to be considered individually. Figure 8C demonstrates such a case, where the presence of an additional Tyr residue does not significantly alter the nature of the optimized geometry of Arg to the first Tyr residue, which possesses a binding energy of −4.0 kcal/mol. This suggests that, upon removal from the protein context, only very strong or crucial interactions might retain optimized geometries that closely resemble those observed in the protein environment, which implies that the various binding modes have synergistic effects on each other.

## Conclusions

In this study, we attempted to clarify the energetic contributions of aromatic–aromatic and cationic–aromatic interactions to protein structures. We aimed to quantify the specificity of these interactions and their sensitivity to geometric variation. Particularly, we aimed to elucidate the versatility of pairwise interactions in proteins and how acknowledged pairwise interactions, such as π–π and hydrogen bonding, are supplemented by cation−π or CH−π interactions. To this end, we performed comprehensive QM calculations of pairwise π–π and cation−π interactions involving Phe, Tyr, Trp, Arg, and Lys, which were categorized using geometric parameters. The QM binding energies of these pairs were overlaid onto a map of the frequency with which each geometry was populated in high-resolution, three-dimensionally resolved protein structures.

Among aromatic–aromatic pairs, we found that the high QM stability calculated for π–π interactions does not correlate with their abundance in structured proteins. Particularly for Phe–Phe pairs, “pure” π–π interactions are not frequently observed in proteins, with mixed π–π and CH−π contributions being preferred. Unexpectedly, we found that Phe–Phe pairs sample the various geometries almost by random chance. Our observation differs from earlier studies that found a high prevalence for pure π–π interactions;^9,53^ however, this reported abundance appears to be a result of the earlier studies using parameters for π–π interactions that were biased toward conformations characterized by short centroid distances. Similarly, for other aromatic–aromatic pairs, stacked orientations were seldom observed, despite their calculated stability, with the exception of pairs involving a Tyr residue. The difference between the behaviors of Phe–Phe and Phe–Tyr pairs underscores the subtle nuances that even a single atom change can introduce into molecular interactions. Our observations may be rationalized when considering the absence of solvent-driven effects when aromatic–aromatic residues are typically buried in proteins. Nevertheless, our results support the potential importance of these interactions in IDPs.

We found that CH−π interactions not only were the most populated but also significantly contributed to stabilizing the global minimum energy conformations of Phe–Tyr and Tyr–Tyr pairs. The similarity of these interactions and cation−π interactions in terms of their geometric and energetic parameters suggests a more prominent role for CH−π interactions than was previously recognized. As standalone interactions, CH−π interactions are of weaker strength than π-stacked ones; however, they are geometrically nonspecific. We find their strength to be weakly dependent on geometric parameters, the identity of the CH donor, and the presence of a solvent.

Unlike π–π pairs, cation−π pair conformations are more populated than can be attributed to random chance and are very attractive, particularly when Arg is involved. For pairs involving Lys, interactions meeting the definitions of both cation−π and CH−π interactions are very favorable; however, these are rarely observed in proteins. Interestingly, cation−π pairs involving Arg favor stacked orientations rather than T-shaped orientations, which is consistent with their generally being exposed to the solvent. Nevertheless, we find that T-shaped conformations are most stable, probably because of the additional H-bonding contribution. This example suggests that inferring the contribution of a specific interaction type solely from its single minimal energy conformation may be misleading. Thus, our approach of calculating multiple conformations from a large sample of protein structures may be beneficial.

Overall, our study illustrates that the aromatic residues Phe, Tyr, and Trp can participate in various types of interactions having comparable strengths. The interplay between these pairwise interactions depends on the nature of the residues and their exact geometries. Our study suggests that current force fields can benefit from re-evaluation of their energetic terms to correctly capture pairwise aromatic interactions. Future studies may include a detailed investigation of His, which has aromatic and cationic properties under conditions of high and low pH, respectively, and can therefore play important roles in π–π and cation−π interactions in proteins.

## Supporting Information Available

Atomistic details of the compounds calculated; higher level QM validation of the QM method employed for representative pairs of amino acids; geometrical parameters used to categorize the interaction types π–π, CH−π, cation−π, and H-bonds; gas-phase binding energies for π–π interacting pairs; cutoff of geometrical angles may lead to different conclusions of populated conformations; CH−π interacting pairs show weak dependence on the donor; gas-phase binding energies for cation−π interacting pairs; preference of Arg’s NH1 atom to form more attractive and frequent interactions with aromatic rings; Arg’s CD atom is frequently observed near the centroid of aromatic rings; and abundance of aromatic and cationic interactions in our neutron-diffraction (ND) data set (PDF)
