# Supplementary material for: Aromatic Residues in Proteins: Re-Evaluating the Geometry and Energetics of π–π, Cation−π, and CH−π Interactions
Source: J Phys Chem B. 2024 Sep 2;128(36):8687–700. doi: 10.1021/acs.jpcb.4c04774 (PMC11403661; doi:10.1021/acs.jpcb.4c04774)
Supplement: Supplementary file 1 — jp4c04774_si_001.pdf [file jp4c04774_si_001.pdf]

Supporting Information:

Aromatic Residues in Proteins: Re-evaluating the Geometry and Energetics of π-π, Cation-π and CH-π Interactions

Rivka Calinsky and Yaakov Levy*

Department of Chemical and Structural Biology
Weizmann Institute of Science
Rehovot, 76100, Israel

*Corresponding author: Yaakov Levy, Department of Chemical and Structural Biology, Weizmann Institute of Science, Rehovot, 76100, Israel; email: Koby.Levy@weizmann.ac.il; Tel: 972-8-9344587

S1 Atomistic details of amino acids subjected to QM calculations

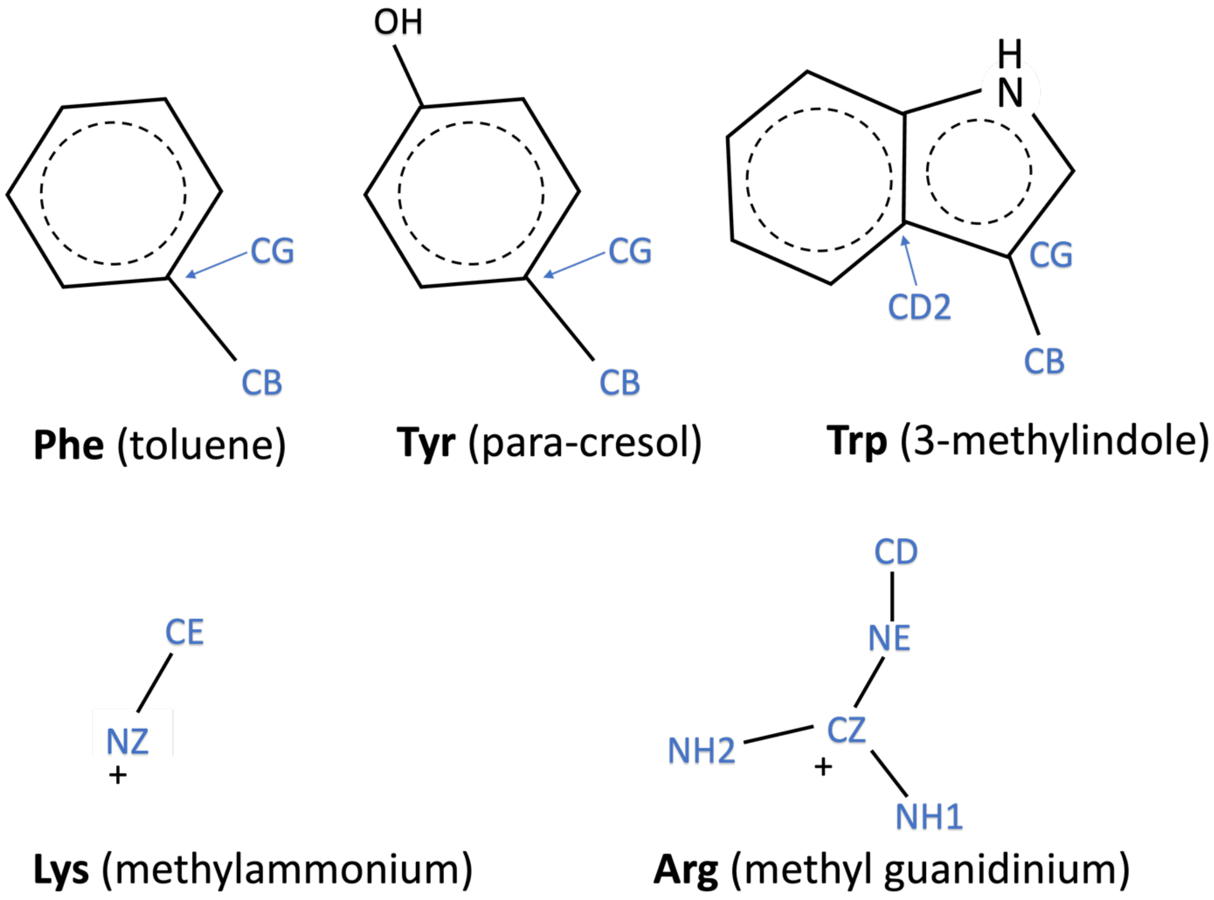

Figure S1. Schematic representations of side chains of amino acids discussed in this study. Blue atoms represent the location of the atoms mentioned in Fig. 1 (main text) as per the PDB naming convention. The corresponding chemical structures used for QM calculations are indicated and referred to as Phe, Tyr, Trp, Lys, and Arg.

S2 Higher level validation of the QM method employed in this work

Table S1: **Validation of the used QM method against the higher level LNO-CCSD(T).**

The LNO-CCSD(T) calculated binding energies ($\boldsymbol{\Delta E^{half}_{LNO-CCSD(T)}}$) using a 'half-counterpoise' method for accuracy [1], derived from both 'raw' and 'Counterpoise-Corrected' (CP) methods to account for Basis Set Superposition Error (BSSE). This method was chosen due to its high performance in terms of accuracy for noncovalent interactions [2]. LNO-CCSD(T) values were calculated using vTight threshold for accuracy, satisfying $\Delta E^{half}_{LNO-CCSD(T)} = \frac{\Delta E_{CP}+\Delta E_{raw}}{2}$, where ΔE_{raw} corresponds to the energy difference of the optimized pair and the separately optimized residues. The ΔE_{CP} corresponds to the difference in energy of the optimized interacting residues and each of the residues where its partner residue is present as a 'ghost' atom (with its basis functions yet no electrons included). The $\Delta E^{raw,\ def2-qzvppd'}_{revDSD-PBE86-D4}$ error of the binding energies correspond to the error from the LNO-CCSD(T) level where the revDSD-PBE-D4 energies were calculated using 'raw' method. The last column corresponds to the fractional error from the higher-level method: $\frac{\Delta E^{raw,\ def2-qzvppd'}_{revDSD-PBE86-D4} - \Delta E^{half}_{LNO-CCSD(T)}}{\Delta E^{half}_{LNO-CCSD(T)}} \cdot 100\%$

**Index**	$\boldsymbol{\Delta E^{half}_{LNO-CCSD(T)}}$	$\boldsymbol{\Delta E^{raw,\ def2-qzvppd'}_{revDSD-PBE86-D4}}$	$\boldsymbol{\Delta E^{raw,\ def2-qzvppd'}_{revDSD-PBE86-D4}}$
	**Energy [kcal/mol]**	**Error**	**%Error**
1	-3.1	0.1	-2.2
2	-4.1	0.2	-5.5
3	-4.8	0.1	-1.7
4	-3.8	0.1	-1.8
5	-4.2	0.0	-0.9
6	-5.3	0.2	-2.9
7	-6.1	0.1	-1.5
8	-4.3	0.3	-6.4
9	-4.2	0.1	-3.1
10	-3.9	0.1	-2.1
11	-5.2	0.2	-3.7
12	-4.3	0.3	-6.8
13	-6.1	0.1	-2.0
14	-6.9	0.2	-2.8
15	-4.4	0.1	-2.4
16	-9.0	0.4	-4.3
17	-11.0	0.2	-1.5
18	-10.1	0.5	-5.0
19	-11.2	0.2	-1.8
20	-9.6	0.2	-1.9
	**Average**	**0.2**	**-3**

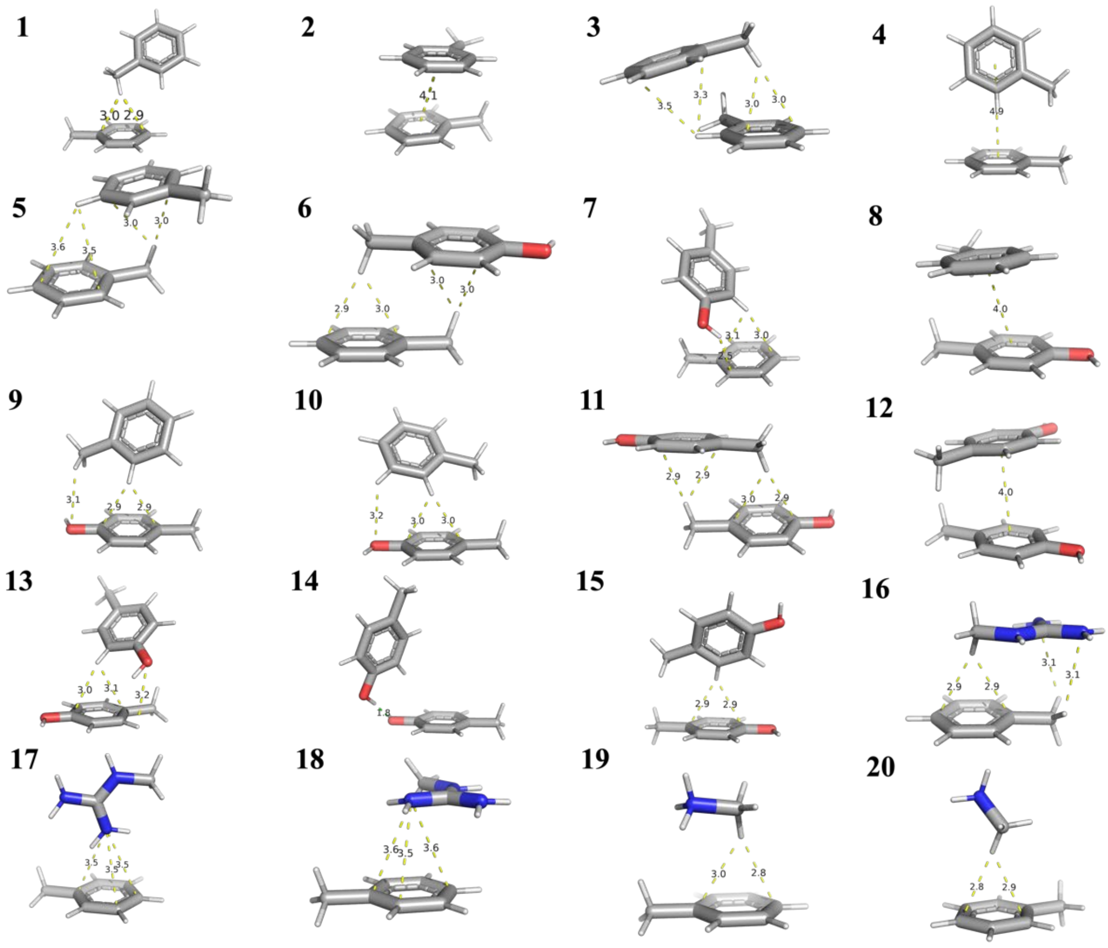

Figure S2. Representative geometries used for QM method validation. The structures are represented according to their index as shown in Table S1.

S3 Geometrical categorization of interaction types

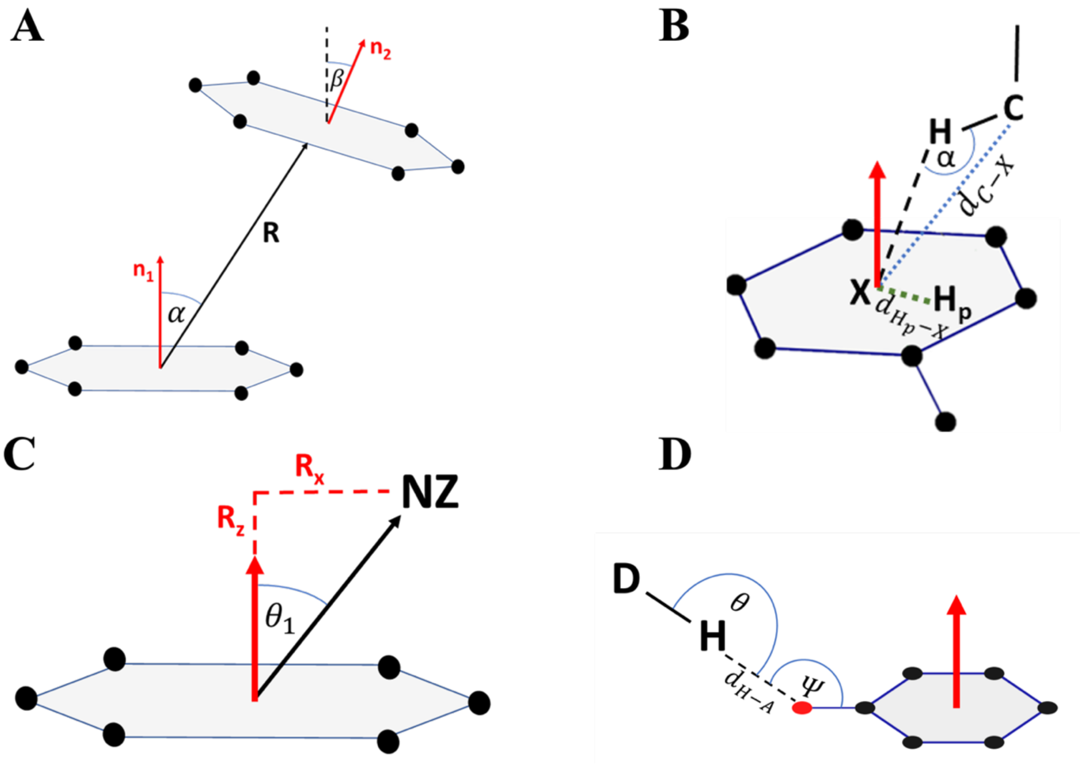

Figure S3. Selected geometrical parameters to classify the four interaction types discussed in this study: π- π, CH- π, Cation- π and H-bonds.

(A) Stacked interactions defined by $R < 5\text{Å}$, $\alpha < 45°$, $\beta < 45°$, where R is the centroids distance of two aromatic rings, α is the angle of R from the first ring's normal $\widehat{n_1}$ and β is the angle of the second's ring's normal $\widehat{n_2}$ from $\widehat{n_1}$. (B) CH-π interaction pairs are chosen according to the Brandl-Weiss system. These are defined with $d_{C-X} < 4.5\text{Å}$, $\alpha > 120°$, $d_{Hp-X} < 1.2\text{Å}$, where d_{C-X} the distance of the closest carbon from the centroid of the aromatic ring, X. d_{Hp-X} is the distance between the closest Hydrogen and the centroid in the ring's projection in the ring's plane (only horizontal component), and α is the angle at the closest Hydrogen atom, used to exclude overlapping π -stacking interactions. (C) Cation- π interactions. Including distance satisfying D≤ 6Å from the center of positive charge (NZ for Lys, CZ for Arg) and the center of the aromatic ring of its pair. Additionally, the projection of this distance on the aromatic ring plane (horizontal component Rx) should satisfy $Rx \leq 2.3\text{Å}$ (D) Hydrogen-bonding. Characterized based on the definitions presented in Baker's work for sp2, for H-bonds distances of $1.4\dot{A} \leq d \leq 2.1\dot{A}$ we chose structures whose angle at the hydrogen atom (ϴ) satisfies $110° \leq \Theta \leq 180°$, and their angle at the acceptor atom Ψ satisfies $70° \leq \Psi \leq 180°$. Similarly, H-bonds for larger distances are screened within: $2.1\dot{A} < d = 2.8\dot{A}$, $80° \leq \Theta \leq 180°$, $60° \leq \Psi \leq 160°$.

S4 Gas-Phase binding energies for π-π interacting pairs

To better represent buried Phe-Phe pairs (and other aromatic pairs), we considered gas-phase calculated binding energies (**Fig. S4**). We found that for these pairs, the binding energy difference between T-shape like and pure stacked interactions' energies is less prominent, where purely stacked interactions are no longer the most energetically favorable, but rather stacked interactions overlapping with the CH-π discussed earlier. Additionally, for solvated pairs, solvent effects are expected to favor stacked configurations by maximizing hydrophobic surface area[23] when considering protein-free energy stability aside from the enthalpic contribution. Hence, the observation of very few stacked conformations can be justified as most of the Phe-Phe pairs in our dataset are buried inside hydrophobic cores.

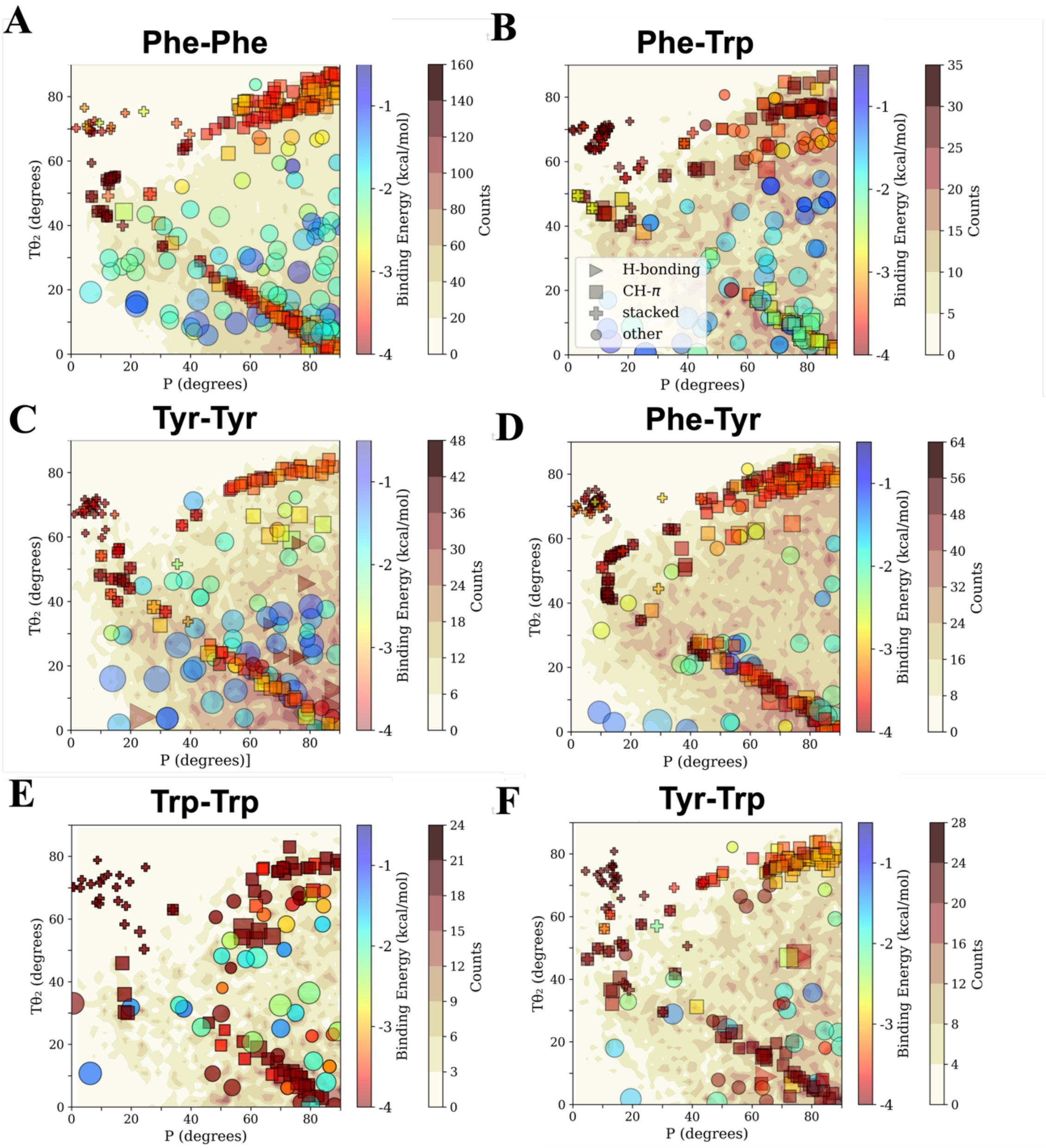

Figure S4. π- π interactions in gas-phase. As described in Fig. 2 (main text) the binding energies (rainbow colorbar) of pairwise Phe interactions calculated for selected geometries and mapped onto a density contour map (white-brown colorbar). (A) For Phe-Phe pairs; and (B) Phe-Trp pairs (C) Tyr-Tyr (D) Phe-Tyr (E) Trp-Trp and (F) Tyr-Trp.

S5 Cutoff of geometrical angles may lead to different conclusions of populated conformations

In our analysis of density maps we observed that shorter distance cutoffs skew towards stacked interactions, as T-shaped distances are typically too long to be detected. As the cutoff increases (see **Fig. S5**), T-shape structures are included, leading to a more normalized distribution of plane angles (around 90° in the range $0° - 180°$). Comparable conclusions can be drawn about the $T\theta_2$ elevation angle for Phe-Phe pairs (**Fig. S6**), which seem to distribute normally about 0° (ranging from $-90°$ to 90°).

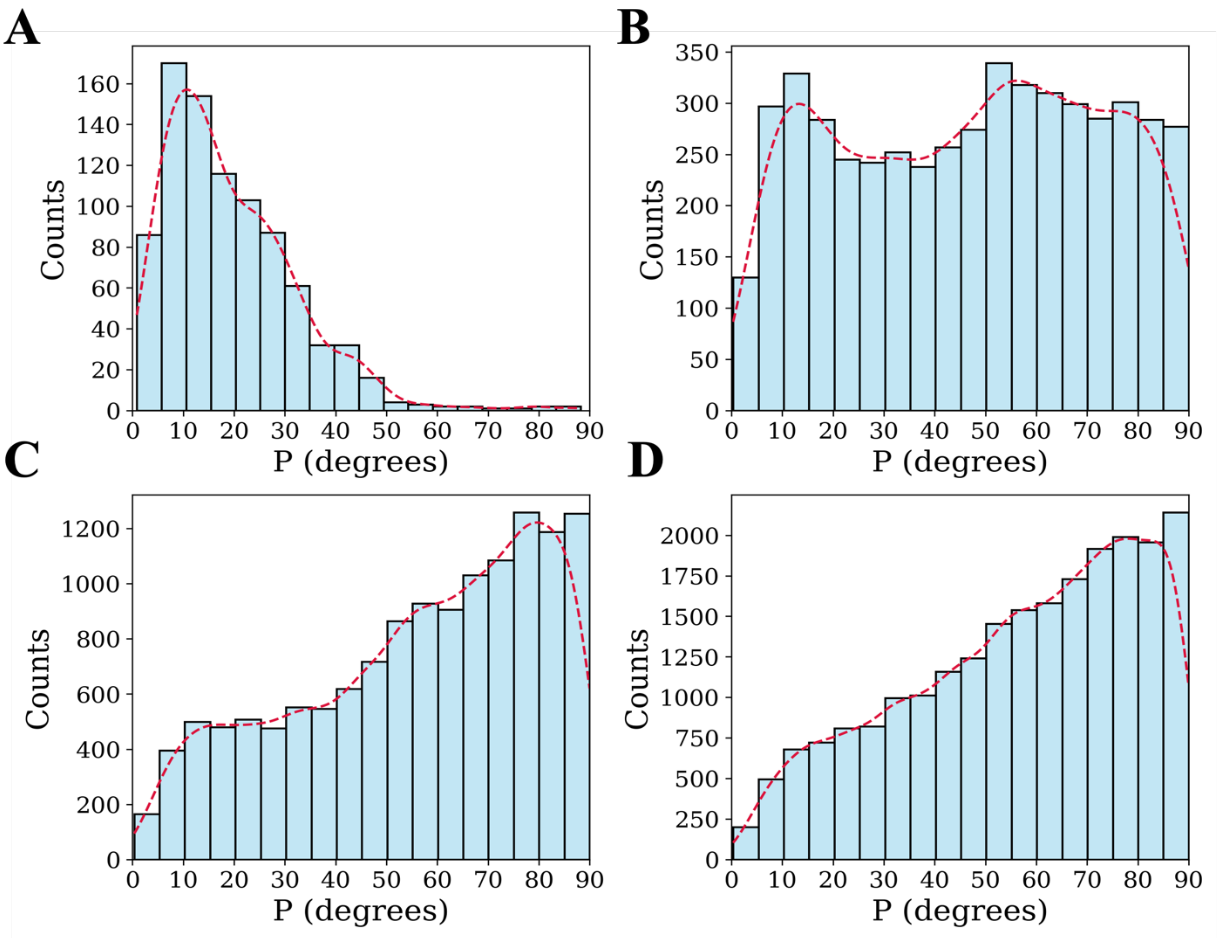

Fig.S5 The dependence of the distribution of P (degrees) on the centroids distance cutoff (D) chosen. (A) D = 4.5 Å (B) D=5 Å (C) D=5.5 Å (D) D=6 Å.

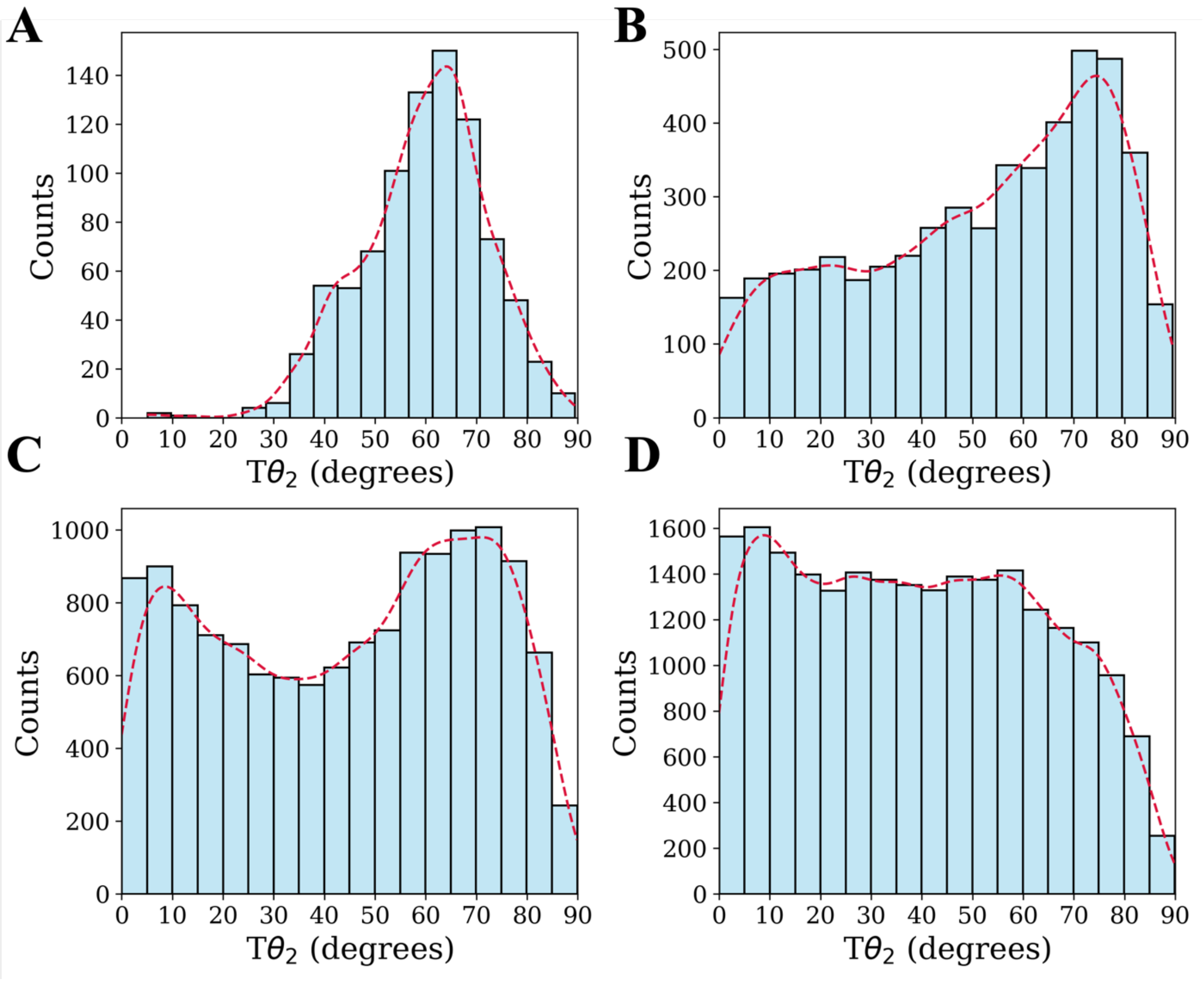

Fig.S6 The dependence of the distribution of $T\theta_2$ (degrees) on the centroids distance cutoff (D) chosen for Phe-Phe pairs. (A) D = 4.5 Å (B) D=5 Å (C) D=5.5 Å (D) D=6 Å.

S6 CH-π interacting pairs show weak dependence on the donor

In the context of heterogenous pairs like Phe-Tyr, it is crucial to distinguish two distinct CH-π interaction groups. In the first group, Phe acts as the hydrogen donor and Tyr serves as the π-acceptor (with $T\theta_2 \geq 45°$), while the second group reverses these roles with Tyr transforming into the hydrogen donor ($for\ T\theta_2 < 45°$). In low-plane angle regions of $10° < P < 20°$ where $40° < T\theta_2 < 55°$, we identified geometries where both entities serve as donors and acceptors, indicating multiple interactions. Specifically, when Trp acts as the acceptor, it can accommodate two cooperative CH-π interactions, even in tilted or T-shaped geometries, attributed to each of its aromatic 5 or 6 membered rings. This cooperativity may provide a plausible explanation for the observed strength increase in Phe-Trp and Tyr-Trp interactions, and for their high standard deviation of distributions when mixing strengths of single and doubly CH-π interactions pairs (**Table 1**).

Table S2: The average strength of CH-π interaction (lower than -1kcal/mol) depending on the C-H donor residue identity, differentiated according to $T\theta_2\ > 45°\ or\ T\theta_2\ < 45°$. When Trp is the acceptor generally two CH- π interactions are observed, one above each of its aromatic rings. When $T\theta_2$ ~$45°$ both residues can serve as donors and acceptors in an off-stacked manner.

	**donor**	**ΔCH-π**	
		**solvent**	**gas-phase**
**Phe-Tyr**	Phe	-3.1±0.3	-3.6±0.4
	Tyr	-3.2±0.3	-3.9±0.7
**Phe-Trp**	Phe	-3.8±0.4	-4.7±0.4
	Trp	-2.9±0.6	-3.6±1.0
**Tyr-Trp**	Tyr	-4.1±0.4	-5.1±0.8
	Trp	-3.4±0.6	-4.1±1.11

S7 Gas-Phase binding energies for cation-π interacting pairs

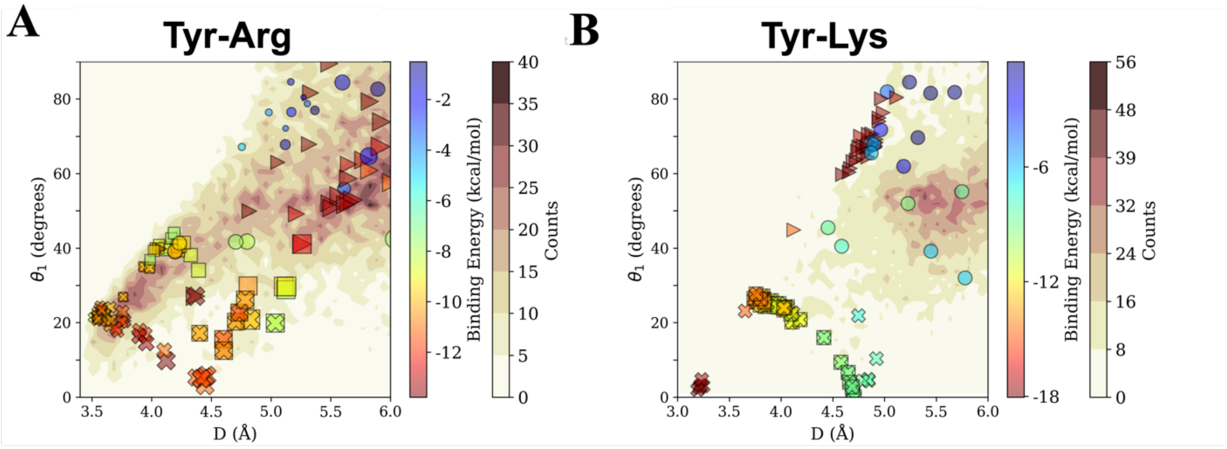

Figure .S7 Tyr's cation- π interactions in gas-phase for (A) Tyr-Arg and (B) Tyr-Lys pairs.

S8 Preference of Arg's NH1 atom to form more attractive and frequent interactions with aromatic rings

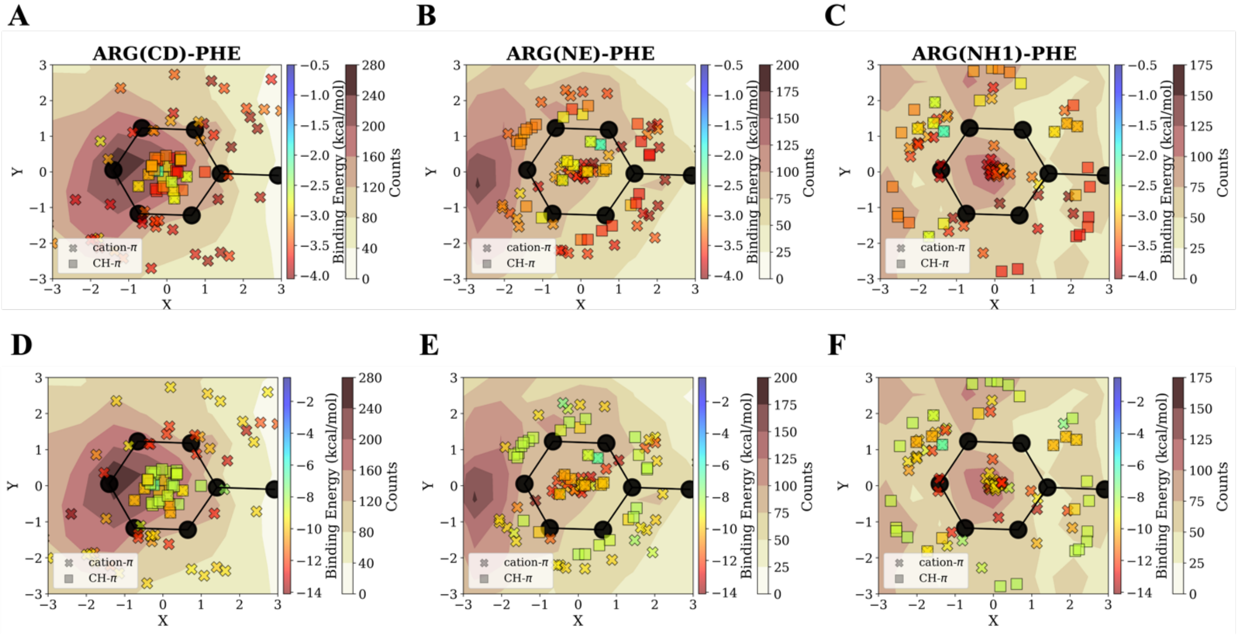

Figure. S8 The dependence of cation-π and CH-π interactions of Arg and Phe on the position of specific atoms in CPCM(water): (A) CD atom (B) NE atom (C) NH1 atom. For vacuum (D) CD atom (E) NE atom (F) NH1 atom.

S9 Arg's CD atom is frequently observed near the centroid of aromatic rings

Investigating the solvated pure CH-π interacting pairs we found Arg-Tyr exhibit energy strengths only slightly weaker, yet comparable, to those of solvated pure cation-π pairs (Table 2 in the main text). These CH-π interactions occur for pairs satisfying the criteria: 4 Å < D < 4.5 Å, 30° < ϴ1 < 45°, and small Θ_2, corresponding to displaced stacked interactions of the CD hydrogen bond atom of Arg with the ring center of Tyr (see **Fig. S2.16**). For higher distances and lower Θ_1 values, the binding energies of solvated CH-π interactions decrease. These latter geometries are less populated, possibly due to steric clashes that may arise when the remaining sidechain atoms of Arg are introduced, considering the T-shape-like orientation of Arg's CD atom facing the Tyr ring (see conformation in Fig. 8B in the main text). Further examination, illustrated in **Fig. S9** for Arg-Phe, suggests that the CD atom is much more frequently observed above the ring's centroid compared to the NE atom (to which CD is directly attached), with the former participating in CH-π interactions. A comparison of CH-π interactions among Phe, Tyr, and Trp with respect to the CD atom is shown in **Fig. S11A-C**, where the CD atom density is consistently observed near the center of the rings in all cases.

For Lys' pairs we observed low abundance of CH-π interactions (**Fig. 4B, D, F**). We found the top part of CH-π interactions (represented along the Θ_1 axis, where ϴ1>15°) corresponds to structures where the Lys's NZ-CE bond runs parallel to the aromatic ring (see Fig.**S2.19**). This is in contrast to configurations in the lower ϴ1 spectrum where the Lys's NZ-CE bond is oriented perpendicularly to the ring (see **Fig.S2.20**). The latter is infrequently observed, possibly due to the potential physical clash when sidechain atoms missing in the QM protein structures are introduced, akin to what is observed in the Tyr-Arg pairing.

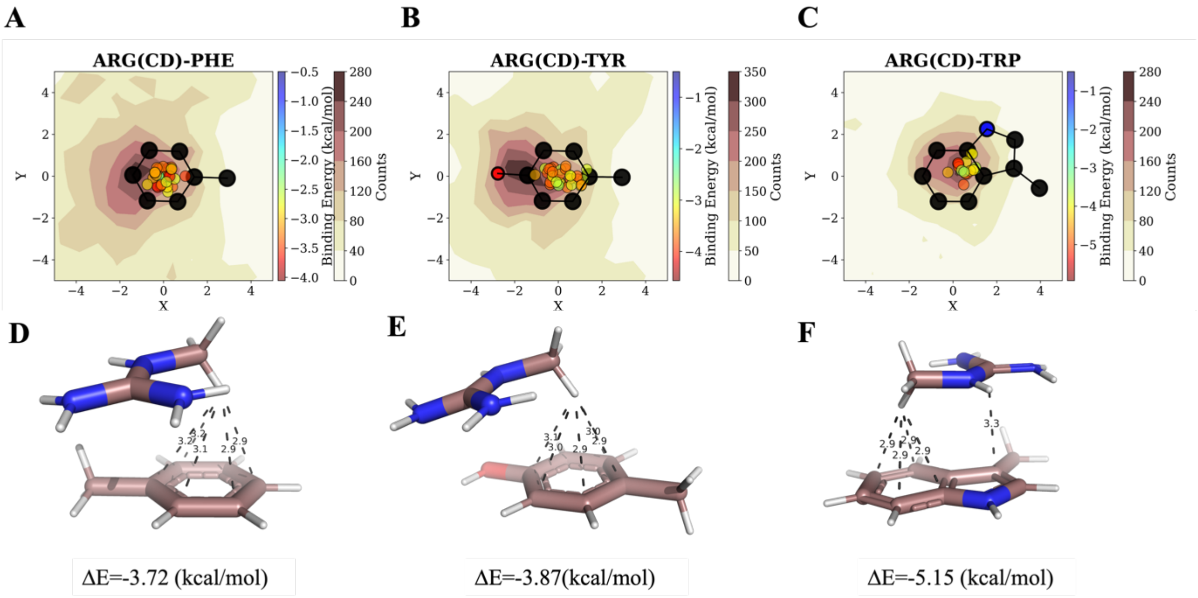

Figure 9) CH-π interactions of Arg according to categories in Fig.S1. Mapping the CD atom of Arg relative to (A) Phe reference (B) Tyr (C) Trp. The markers show only QM pairs categorized as CH-π interacting. Representative conformations of lowest energy CH-π (D) Phe (E) Tyr (F) Trp.

S10 Abundance of aromatic and cationic interactions in our Neutron-Diffraction (ND) dataset

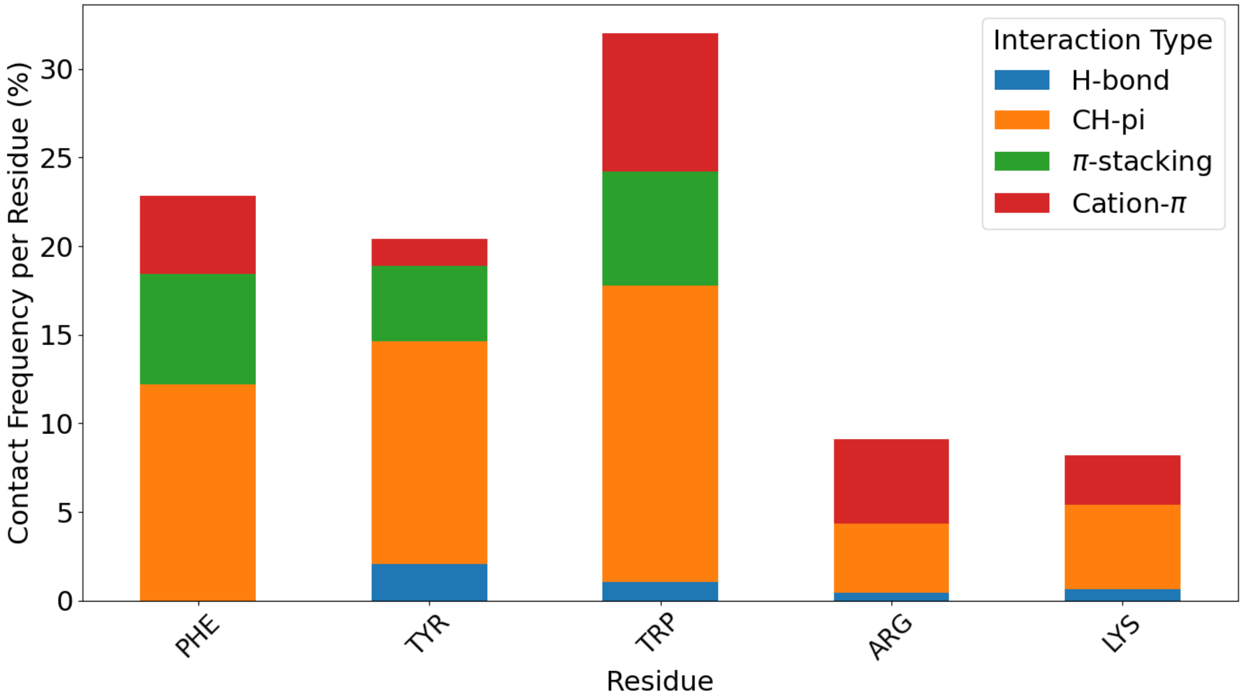

Figure 10) Distribution of interaction types normalized by residue occurrence for aromatic (PHE, TYR, TRP) and cationic (ARG, LYS) residues. The stacked bar chart illustrates the relative frequency (%) of various molecular interactions between these five residues: H-bonds, CH-π, π-stacking, and cation-π interactions. The frequencies are calculated by normalizing the count of each interaction type by the total number of occurrences of the respective residue in the dataset (PHE: 657, TYR: 588, TRP: 281, ARG: 715, LYS: 941).

References:

[1] Comparing Counterpoise-Corrected, Uncorrected, and Averaged Binding Energies for Benchmarking Noncovalent Interactions. Lori A. Burns, Michael S. Marshall, and C. David Sherrill. Journal of Chemical Theory and Computation 2014 10 (1), 49-57
DOI: 10.1021/ct400149j

[2] Emmanouil Semidalas, Golokesh Santra, Nisha Mehta, Jan M. L. Martin; S66 noncovalent interactions benchmark re-examined: Composite localized coupled cluster approaches. AIP Conference Proceedings 23 November 2022; 2611 (1): 020016.
https://doi.org/10.1063/5.0119282
